# Predicting the Role of IL-10 in the Regulation of the Adaptive Immune Responses in *Mycobacterium avium* Subsp. *paratuberculosis* Infections Using Mathematical Models

**DOI:** 10.1371/journal.pone.0141539

**Published:** 2015-11-30

**Authors:** Gesham Magombedze, Shigetoshi Eda, Judy Stabel

**Affiliations:** 1 National Institute for Mathematical and Biological Synthesis, University of Tennessee, Knoxville, Tennessee, 37996–1527, United States of America; 2 MRC Centre for Outbreak Analysis & Modelling, Department of Infectious Disease Epidemiology, Imperial College London, London, United Kingdom; 3 Department of Forestry, Wildlife, and Fisheries, University of Tennessee, Knoxville, Tennessee, 37996–1527, United States of America; 4 USDA-ARS, National Animal Disease, Ames, Iowa, 50010, United States of America; Indian Institute of Technology Delhi, INDIA

## Abstract

*Mycobacterium avium* subsp. *paratuberculosis* (MAP) is an intracellular bacterial pathogen that causes Johne’s disease (JD) in cattle and other animals. The hallmark of MAP infection in the early stages is a strong protective cell-mediated immune response (Th1-type), characterized by antigen-specific *γ*-interferon (IFN-*γ*). The Th1 response wanes with disease progression and is supplanted by a non-protective humoral immune response (Th2-type). Interleukin-10 (IL-10) is believed to play a critical role in the regulation of host immune responses to MAP infection and potentially orchestrate the reversal of Th1/Th2 immune dominance during disease progression. However, how its role correlates with MAP infection remains to be completely deciphered. We developed mathematical models to explain probable mechanisms for IL-10 involvement in MAP infection. We tested our models with IL-4, IL-10, IFN-*γ*, and MAP fecal shedding data collected from calves that were experimentally infected and followed over a period of 360 days in the study of Stabel and Robbe-Austerman (2011). Our models predicted that IL-10 can have different roles during MAP infection, (i) it can suppress the Th1 expression, (ii) can enhance Th2 (IL-4) expression, and (iii) can suppress the Th1 expression in synergy with IL-4. In these predicted roles, suppression of Th1 responses was correlated with increased number of MAP. We also predicted that Th1-mediated responses (IFN-*γ*) can lead to high expression of IL-10 and that infection burden regulates Th2 suppression by the Th1 response. Our models highlight areas where more experimental data is required to refine our model assumptions, and further test and investigate the role of IL-10 in MAP infection.

## Introduction

Johne’s disease (JD) is a mycobacterial disease of ruminants which has a significant global economic impact [1]. It is caused by *Mycobacterium avium* subsp. paratuberculosis (MAP) bacteria and infection primarily occurs in the intestine. End-stage MAP infection is normally characterised by persistent and progressive diarrhea, weight loss, debilitation and eventually death. However, disease manifestations following exposure to MAP can be highly variable in degree of severity. An intriguing characteristic that can be associated with intracellular mycobacterial pathogens is their ability to shift between avirulent and virulent metabolic states within the host [2–4]. This attribute may be responsible for the varying degree of insult to host immunity and drive disease disparately in different animals.

After exposure to the MAP pathogen, disease progression is slow with infected animals remaining asymptomatic for extended periods of time. Typically, Th1-type responses (cellular immunity) with predominant secretion of IFN-*γ* occur soon after MAP infection [5]. As infection progresses, the Th1 response is reported to decline in some studies [5–7] and is supplanted by a Th2-type response (humoral immunity). The Th2-type immune response becomes more dominant in the clinical stages of the disease. This is known as the classical Th1/Th2 immune response switch reported to be common in ruminants infected with MAP [6–9]. In addition, Th1 and Th2 immune response trends also correlate with pathologic lesions [6, 10–12]. In sheep MAP infection, paucibacillary lesions have been correlated with a stronger cell-mediated immune response than those with multibacillary lesions [5, 13]. A similar trend of higher levels of Th2 type cytokines IL-4, IL-10, IL-2 were observed in cattle with multibacillary lesions [14].

The immune response associated with MAP infection is complex and currently it is not completely understood. How a strong Th1-mediated (IFN-) *γ* response in the early stages of infection is subsequently lost and replaced with a Th2 (antibody) response [6, 7, 15] is still to be clearly elucidated. Recently, it has become a subject of research and discussion that cytokines with an immunosuppressive effect on Th1 responses, in particular IL-10 and tranforming growth factor (TGF)-*β*, may mediate this change [7, 13, 16]. In the study of de Silva et al. [13], IL-10 was shown to be more significantly expressed in animals with no lesions or with paucibacillary lesions than in animals with multibacillary lesions. The expression of IL-10 in the peripheral blood mononuclear cells was thought to be reflective of the early response at the site of MAP infection. The success against immunopathology of mycobacterial pathogens is delicate and requires a balance between the innate and the adaptive response to limit tissue destruction by effector mechanisms. IL-10 is one cytokine that is considered to play a major role in maintaining this balance [17].

IL-10 is an anti-inflammatory cytokine with a crucial role in preventing inflammatory and autoimmune pathologies [17]. Although the absence of IL-10 leads to better clearance of some pathogens [17], the absence of IL-10 can be accompanied by immunopathology that is detrimental. IL-10 was initially described as a Th2-type cytokine, but further evidence suggested that the production of IL-10 was associated with regulatory T (Treg) cells [17–19]. It is now known that the expression of IL-10 is not specific to Th2 or Treg cells, but to a much broader array of cells including CD4+ T cells (Th1, Th2, Th17), CD4+ Treg, CD8+ T cells, γδ T cells, B cells, and macrophages [17, 19]. An understanding of how IL-10 expression is regulated in different pathologies is of importance. There is accumulating evidence that IL-10 may be important in MAP infection yet the literature suggests two conflicting roles. Buza et al. [16] showed a suppressive role of IL-10 on IFN-*γ* responses which correlated with observed immunopathology of the disease. It was suggested that the suppression of MAP-specific IFN-*γ* and cell proliferative responses may lead to decreased systemic and localized cell-mediated immune responses resulting in suboptimal killing of MAP and subsequently resulting in chronic infection associated with the disease. In contrast, other reports [7, 20] showed that early expression of IFN-*γ* and IL-10 were associated with protection. Though IL-10 has potent immunosuppressive effects it also enhances survival and differentiation of B cells [21]. It has been previously shown that sheep with high numbers of B cells conferred resistance to MAP infection [22]. A possible rapprochement between these different plausible effects is that at tissue level, IL-10 tightly controls the immune response to minimise tissue damage, while at the peripheral level, both IFN-*γ* and IL-10 could have a protective role, since IFN-*γ* is associated with a strong host cell mediated immune response and IL-10 allows for disease resistance and decreased likelihood of infection [7, 20].

In this present study we developed mathematical models to investigate potential roles of IL-10 in the regulation of the adaptive immune responses in MAP infection. We tested the developed models with MAP experimental infection data from the study of Stabel and Robbe-Austerman [15] where calves were challenged with MAP and several immune variables were measured (IL-10, IL-4, IFN-*γ* and MAP CFUs. We used the model to predict how IL-10 regulates the Th1 and Th2 responses, and how these adaptive immune responses are correlated with MAP CFU fecal shedding. Due to limited experimental data in this area, we designed our study to stimulate critical thinking, tease out questions and derive insights that will pave way for new experimental studies that will be carefully designed to elucidate the functions of IL-10 in MAP infections.

## Methods and Materials

### Experimental Data

We obtained calf MAP infection data from the study of Stabel and Robbe-Austerman [15]. Apart from the data reported in this study, we obtained additional data from the authors (data not previously published). The data include IL-4 secretion and MAP bacteria shedding patterns of the infected animals that developed Johne’s disease. Although the study included 5 different infection groups, we chose to incorporate only data from the oral mucosa group here. This selection is based on substantial levels of MAP bacteria (excreted bacteria) that was observed for animals within this group. This group of calves (n = 3) were inoculated by feeding milk replacer containing 2.6 x 10^6^ live MAP obtained by scraping the ileal mucosa of a clinically infected cow. Calves were dosed on days 0, 7, and 14 of the study. All procedures performed on the animals were approved by the Institutional Animal Care and Use Committee (National Animal Disease Center (NADC), Ames, Iowa. Intracellular IFN-*γ*, IL-4, and IL-10 were measured from peripheral blood monoclear cells as previously described [15]. MAP bacteria CFUs were measured from feces using standard culture method on Herrold’s egg yolk medium [15].

### Mathematical modelling approaches

**Fig 1:**
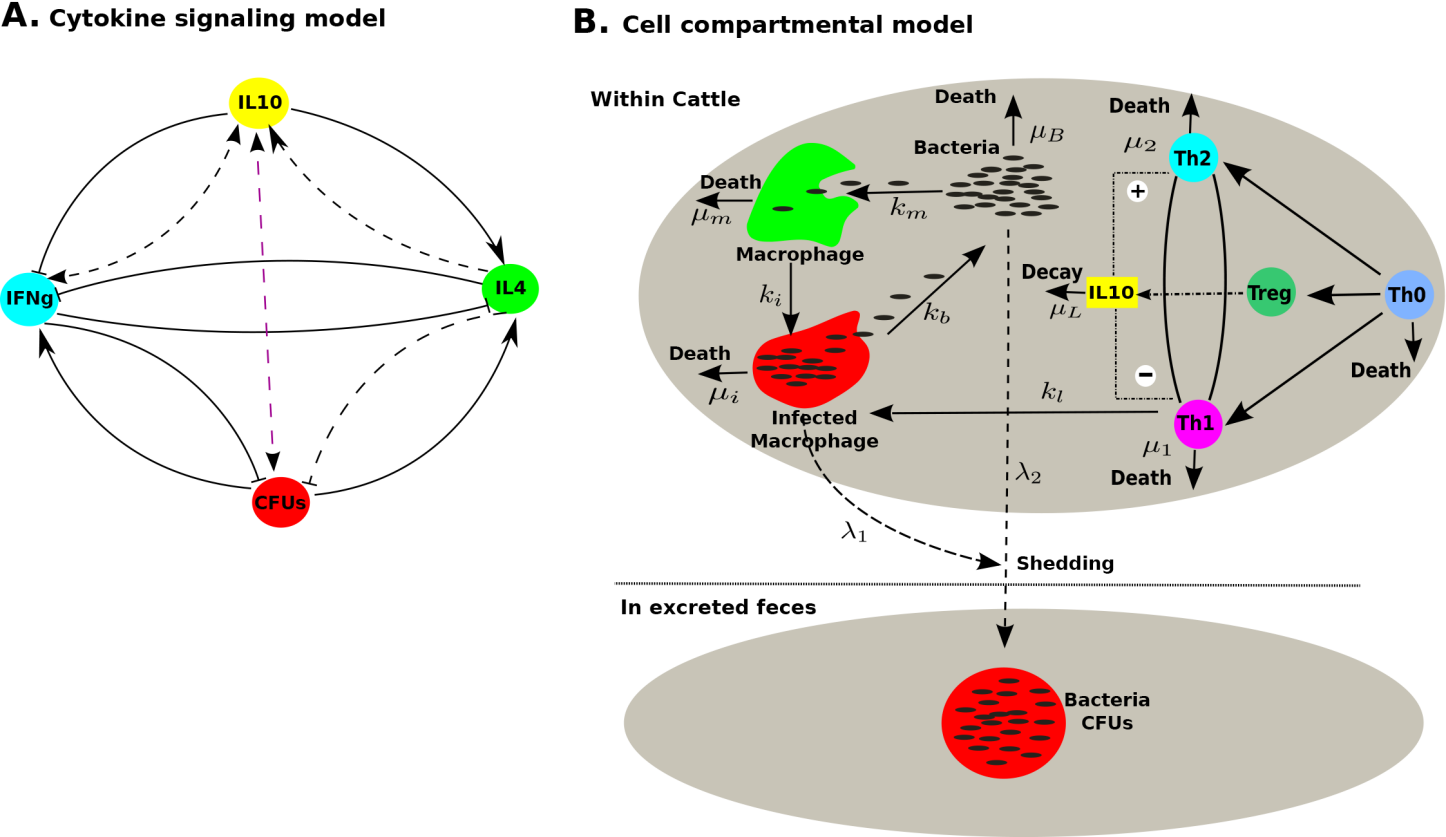
Model cartoons. **A**) A cytokine signaling model diagram. IFN-*γ*, IL-4, IL-10 are assumed to interact together and in-turn influence immune responses and these potentially affect MAP CFU shedding. The model assumes that IL-10 can take multiple functions for instance, they can be assumed to enhance IL-4 production or can be assumed to inhibit IFN-*γ* production. Further production of IL-10 can be assumed to be positively correlated with the amount of CFUs. Also, the amount of excreted CFUs can be correlated with the expressed Th1 and Th2 responses and as well as by IL-10. Arrowed lines represent stimulation, lines with a flat end represent suppression/inhibition. Broken arrowed lines represent probable mechanisms without clear support from biological evidence that may represent a probable inhibition or stimulation. **B**) A cell compartmental model diagram. Macrophages remove/kill free bacteria at rate *k*_*m*_ and get infected at rate *k*_*i*_ giving rise to infected macrophages. Uninfected and infected macrophages have death rates *μ*_*m*_ and *μ*_*i*_, respectively. Infected macrophages burst at rate *k*_*b*_ and they release *N*_*o*_ bacteria at the same time. A Th1 response is assumed to kill infected macrophages at rate *k*_*l*_. IFN-*γ* and IL-4 are assumed to be Th1 and Th2 surrogates. Treg cells are assumed to produce IL-10 and this is captured indirectly through the population of infected macrophages. IL-10 is assumed to either enhance IL-4 production or suppress IFN-*γ* production. Both the population of infected macrophages and free bacteria are assumed to be the source of bacteria excreted in feces at rates *λ*_1_ and *λ*_2_, respectively.

We developed and implemented two different modelling frameworks to closely examine and understand the experimental data. First, we developed a cytokine signalling model to analyse and predict the interactions between the measured variables, the cytokines IFN-*γ*, IL-4, IL-10 and how these correlate with CFU/ bacteria patterns in the excreted faeces (Fig 1A). We use the model to explore how IL-10 regulates the expression of IFN-*γ* and IL-4 and in turn how CFU shedding is altered. Second, we developed a model that realises cell interactions within the host and bacteria outside the host (in the excreted faeces). The model then links the within host compartment and the outside of host compartment (Fig 1B). This model is centred on the assumption that the cytokines, IFN-*γ* and IL-4 (measured in the experiment) are strong correlates (or surrogates) for Th1 and Th2 immune responses [8, 23–25]. The CFU measurements are taken as the cultivated (cultured) population of MAP bacteria from the gut excrement. The model is then used to investigate how IL-10 regulates the Th1 and Th2 responses and how this translates to the observed CFU shedding kinetics.

### Cytokine signalling and bacteria shedding model

This model considers only four variables, three cytokines (IFN-*γ*, IL-4 and IL-10) and MAP bacteria CFUs. This model seeks to explain mechanistic interactions between IL-4, IFN-*γ* and the regulatory role of IL-10 that correlates with the fecal shedding patterns of the infected calves. The parameters *α*_*i*_(*i* = 1−4) and *β*_*i*_(*i* = 1−4) represent the production/stimulation and decay of the biological variables represented by the variables *IFN*, *IL*_4_, *IL*_10_, and *CFU*, respectively. The parameters *h*_*i*_(*i* = 1−2) represent the inhibitory/suppression effects of these biological variables (cytokines) on each other. And *γ*_1_ and *γ*_2_ represent the elimination of bacteria by IFN-*γ* and IL-4 associated responses, respectively. MAP bacteria are assumed to expand at rate *r*.

$$\begin{array}{l} \frac{dIFN}{dt}=\alpha_{1}\frac{CFU}{1+h_{1}IL_{10}IL_{4}}-\beta_{1}IFN\\ \frac{dIL_{4}}{dt}=\alpha_{2}\frac{CFU.IL_{10}}{1+h_{2}IFN}-\beta_{2}IL_{4}\\ \frac{dIL_{10}}{dt}=\alpha_{3}IL_{4}.CFU-\beta_{3}IL_{10}\\ \frac{dCFU}{dt}=(\alpha_{4}IL_{10}+r)CFU-\gamma_{1}IFN.CFU-\gamma_{2}IL_{4}CFU-\beta_{4}CFU \end{array}$$ (1)

The system of Eq (1) represent an example mechanistic mathematical model with terms that explain the biological interactions between IFN-*γ*, IL-4, IL-10 and CFUs as described by Fig 1A. This model only explains some of the possible interactions between these biological variables, however, through model fitting the model can be simplified further or more terms can be added in a way to find the best mechanistic terms that explains the data. Predicted models that best explains the data are given in the supporting information file (S1 Text) and the corresponding estimated parameters are given in Table 1.

**Table 1:** Estimated parameters for the signaling models. Parameters shown were those that were varied during fitting between the models. Dash (-) means a parameter was fixed during fitting and they have the same values between the models, that is *h*_*i*_ (*i* = 1 − 2) = 10.0, *A*_*i*_ (*i* = 1 − 3) *α*_*i*_(*i* = 1 − 4) = 0.0033, *β*_*i*_(*i* = 1 − 4) = 0.015, *γ*_*i*_(*i* = 1 − 2) = 0.015. The system of equations/models (S1), (S2) and (S3) in S1 Text were used to generate the fits (Fig 2) and in estimating the parameters. Priors for *α*’s and *β*’s were set between 0–1, while for *h*_*i*_’s between 0–10.

Parameter	*α*_1_	*α*_2_	*α*_3_	*α*_4_	*β*_1_	*β*_2_	*β*_2_	*β*_4_	*h*_1_	*A*_3_	*r*
Calf A (95%CIs)	0.9552(0.873–0.999)	0.8841(0.803–0.972)	0.00074(0.0007–0.0008)	0.00003(0.00002–0.00004)	0.0051(0.0045–0.0055)	0.0111(0.0097–0.0126)	0.0082(0.0074–0.0092)	0.000145(0.0001–0.00024)	1.2(1.1–1.3)	-	-
Calf B (95%CIs)	0.033(0.018–0.048)	0.046(0.025–0.059)	-	0.073(0.040–0.098)	0.0029(0.001–0.0048)	-	0.015(0.008-.025)	-	-	0.00053(0.0001–0.001)	-
Calf C (95%CIs)	0.0159(0.0152–0.0168)	0.0208(0.0201–0.0216)	-	0.0009(0.00076–0.00108)	0.0027(0.00259–0.00277)	-	0.0167(0.0153–0.0179)	0.7688(0.7348–0.8038)	-	-	0.8436(0.8043–0.8819)

### Cell interaction and bacteria shedding compartmental model

In our second approach we devised a model based upon our previous within-host MAP infection model [26] which explains the interactions of immune cells, phagocytes, and MAP bacteria in the small intestines (in the peyer’s patches). In this new model we focused on the role of IL-10 in the regulation of Th1 and Th2 specific responses and how these correlates with MAP bacteria excreted in the feces. The model assumes two different compartments, (i) the within cattle compartment, in which interaction of MAP bacteria (*B*), macrophages (*Mϕ*, target cells and *I*_*m*_, infected macrophages), CD4 T cell subsets (Th0 precursors, Th1 (*T*_*h*1_)and Th2 (*T*_*h*2_) cells), and IL-10 is assumed to take place in the small intestine tissue, and (ii) the outside cattle compartment (excreted feces in the environment), in which the amount of excreted MAP bacteria is reflected by measured CFUs (*see* Fig 1B).

Uninfected macrophages (*Mϕ*) are recruited to the site of infection at a rate of *σ*_*m*_ cells per day, die at a rate *μ*_*m*_ and become infected through ingesting free MAP bacteria (*B*) at rate *k*_*i*_ per day. Ingested bacteria can be killed by macrophages at rate *k*_*m*_, while macrophages that become infected eventually rupture at rate *k*_*b*_ per day, releasing *N*_0_ bacteria in the process and can die at a rate of *μ*_*I*_ per day. Th1 cells remove infected macrophages at rate *k*_*l*_ and kill intracellular bacteria in the process. A fraction *λ*_1_ of infected macrophages is lost from the within host compartment to the outside (excreted feces) compartment and also a fraction *λ*_2_ of free bacteria is lost to the excreted feces compartment. These two fractions are assumed in this model as the source for MAP bacteria (CFUs determined by fecal culture tests) in the excreted feces. MAP bacteria in feces are assumed to have a death rate of *μ*_*CF*_ per day. However, mechanisms of MAP bacteria excretion from the within cattle compartment are still unclear, there are speculations that infected macrophages translocate from the lamina propria and the peyer’s patches through the epithelial lining then get leaked into the intestine fluid/flora leading to their excretion. The model assumes that MAP (CFU) observed in feces should be reflective of the host-pathogen interactions taking place within the cattle since high MAP shedding is reported to be correlated with disease progression [6, 7]. However, it still remains to be verified if the amount of MAP CFUs in feces is in general a true indicator of MAP bacteria in the intestine microbiota. The study of Mentula et al. [27] showed that there is a significant difference between microbes in intestine and feces microbiotas.

Th0 cells differentiate into either Th1 or Th2 cells [23, 28]. In our model, MAP-specific naive CD4 T cells (Th0) are recruited to the infection site at a rate *σ*_*O*_ from the thymus and decay at rate *μ*_0_. To model cellular (Th1) and antibody (Th2) immune responses to MAP we make the simplest assumption that Th1 response is driven by the density of infected macrophages *I*_*m*_, and Th2 response is driven by the density of extracellular bacteria, *B*. There is growing evidence that IL-10 in MAP infection are produced by regulatory T cells (Treg/Tr-1 cells) [24, 29]. We assume that Th0 cells also differentiate into Treg cells, however we do not add an equation for Treg cells in order to keep the model simple.

We make a simple assumption that IL-10 can be produced in proportion to the population of infected macrophages [30] with the term, ($\theta_{3}\delta_{T_{reg}}I_{m}Th_{0}$), where $\delta_{T_{reg}}$ is their rate of production per day. However, the role of IL-10 alternates between being suppressive or supportive toward the expression of both Th1 and Th2 cells, respectively. Taking the assumption that IL-10 will suppress Th1 response while on the other hand it can induce the expression of Th2 cells, in the model the IL-10 Th1 suppression is modeled with the term $\frac{\theta_{1}\delta_{m}I_{m}T_{h_{0}}}{1+a_{1}IL_{10}}$ and the induction of Th2 cells by IL-10 is modelled with the term $\theta_{2}\delta_{B}BT_{h_{0}}IL_{10}$ where *θ*_1_ and *θ*_2_ are the parameters determining the magnitude of clonal expansion of the Th1 and Th2 responses, while *δ*_*m*_ and *δ*_*B*_ are the differentiation rates of Th1 and Th2 cells from Th0 cells, respectively. The parameter, *a*_1_, is the suppressive effect of IL-10 on Th1 cells. Th1 and Th2 effectors decay at rates *μ*_1_ and *μ*_2_, respectively, while IL-10 decays at a rate of *μ*_*L*_ per day. The system of Eq (2) model these biological processes and Fig 1B gives a schematic represenation of the interactions.

$$\begin{array}{l} \frac{dM_{\phi }}{dt}=\sigma_{m}-k_{i}M_{\phi }B-\mu_{m}M_{\phi },\\ \frac{dI_{m}}{dt}=k_{i}M_{\phi }B-k_{b}I_{m}-k_{l}I_{m}T_{h_{1}}-\mu_{I}I_{m}-\lambda_{1}I_{m},\\ \frac{dB}{dt}=N_{o}k_{b}I_{m}-k_{i}M_{\phi }B-k_{m}M_{\phi }B-\mu_{B}B-\lambda_{2}B,\\ \frac{dTh_{0}}{dt}=\sigma_{o}-\delta_{m}I_{m}T_{h_{0}}-\delta_{T_{reg}}I_{m}T_{h_{0}}-\delta_{B}BT_{h_{0}}-\mu_{0}T_{h_{0}}.\\ \frac{dT_{h_{1}}}{dt}=\theta_{1}\delta_{m}I_{m}T_{h_{0}}(\frac{1}{1+a_{1}I_{L_{10}}})-\mu_{1}T_{h_{1}},\\ \frac{dT_{h_{2}}}{dt}=\theta_{2}\delta_{B}BT_{h_{0}}I_{L_{10}}-\mu_{2}T_{h_{2}},\\ \frac{dI_{L_{10}}}{dt}=\theta_{3}\delta_{T_{reg}}I_{m}T_{h_{0}}-\mu_{L}I_{L_{10}},\\ \frac{CFU_{s}}{dt}=\lambda_{1}N_{o}I_{m}+\lambda_{2}B-\mu_{CF}CFU_{s}. \end{array}$$ (2)

### Model parameters

Parameters for the model (system of Eq (2)) are given in Table 2. In Table 3, parameters that were estimated through model fitting to data are given. The values of the parameters that were fixed durig model fitting were derived using information from literature sources [25, 26, 31–35].

**Table 2:** Model parameters. We list parameters that have been derived using information from the following literature sources [6,25–26, 31–35].

Name	Definition	Dimension	Range	Value
*σ*_*m*_	Macrophage supply	cell/mm^3^/day	8.0–10.0	10.0
*σ*_*O*_	Th0 supply	cell/mm^3^/day	0.1–1.0	0.1
*μ*_*m*_	Macrophages death rate	day^−1^	0.11–0.025	0.02
*μ*_*I*_	Infected macrophages death rate	day^−1^	0.11–0.025	0.02
*μ*_*B*_	Bacteria death rate	day^−1^	0–1.0	0.03
*μ*_0_	Th0 decay/death rate	day ^−1^	0.1–0.03	0.01
*μ*_1_	Th1 decay/death rate	day ^−1^	0.1–0.03	0.03
*μ*_2_	Th2 decay/death rate	day ^−1^	0.01–0.02	0.02
*μ*_*L*_	IL-10 decay rate	day ^−1^	0.01–0.02	0.02
*μ*_*CF*_	CFU decay rate	day ^−1^	0.01–0.02	0.02
*k*_*i*_	Macrophage infection rate	mm^3^/cell/day	0-10^−2^	0.002
*k*_*m*_	Bacteria removal by macrophages	mm^3^/cell/day	0-10^−4^	0.000125
*k*_*b*_	Infected macrophages burst rate	day^−1^	0-10^−4^	0.00075
*k*_*l*_	Th1 lytic effect	mm^3^/cell/day	0.0–0.2	0.00004
*N*_*o*_	Burst size		80.0–100.0	100.0
*θ*_1_	Th1 cells clonal expansion	-	1.0–9000.0	1000.0
*θ*_2_	Th2 cells clonal expansion	-	1.0–9000.0	1000.0
*θ*_3_	Treg cells clonal expansion	-	1.0–9000.0	500.0
*δ*_*m*_	Th0 differentiation into Th1 cells	mm^3^/cell/day	0.0–1.0	0.01
*δ*_*B*_	Th0 differentiation into Th2 cells	mm^3^/cell/day	0.0–1.0	0.01
$\delta_{T_{reg}}$	Th0 differentiation into Treg cells	mm^3^/cell/day	0.0–1.0	0.01
*λ*_1_	Bacteria shedding (from *I*_*m*_)	day^−1^	0.0–1.0	0.002
*λ*_2_	Bacteria shedding (from *B*)	day^−1^	0.0–1.0	0.002
*a*_1_, *a*_2_	IL10- Th1 differentiation inhibition	day^−1^	0.0–1.0	0.005
*h*_1_, *h*_2_	Th2/Th1 differentiation inhibition	day^−1^	0.0–1.0	0.005

**Table 3:** Estimated parameters. Estimated parameters through fitting the cell compartmental model to the calf experimental data. Where there is a dash (-) it means the parameter was not fixed at its baseline values. For each estimated parameter a 95% credible intervals (CIs) is given. Priors for these parameters are given as parameter ranges in Table 2. During model fitting, all models initial conditions were set at: uninfected macrophages *M*(0) = 500.0, infected macrophages *I*_*m*_ (0) = 0.0, free bacteria *B*(0) = 1.0, naïve T cells *Th*_0_(0) = 0.1). For model fitting where these initial conditions were estimated see S3 Fig.

Parameter	*k*_*i*_	*k*_*b*_	*λ*_1_	*δ*_*m*_	*δ*_*B*_	$\delta_{T_{reg}}$	*θ*_2_	*p*_2_	*u*_1_	*u*_2_	*a*_1_	*a*_2_	*u*_*cf*_
**Calf A (95%CIs)**	0.0025 (0.0022–0.0029)	0.0026(0.0015–0.0026)	0.99(0.80–1.00)	0.031(0.027–0.040)	0.0287(0.025–0.030)	0.99(0.80–1.00)	348.21(335.08–354.93)	-	-	-	-	-	-
**Calf B (95%CIs)**	0.2156(0.1636–0.2717)	0.0187(0.0108–0.0174)	0.175(0.060–0.317)	0.2426(0.1028–0.2436)	0.0251(0.0212–0.0397)	-	-	0.00397(0.0028–0.0054)	0.200(0.100–0.2535)	-	-	-	0.0100(0.0030–0.0111)
**Calf C (95%CIs)**	0.7940(0.5519–1.000)	0.0083(0.0016–0.0145)	0.4386(0.1638–0.8260)	-	0.0315(0.0052–0.0663)	-	-	-	0.0427(0.0097–0.0642)	0.0012(0.0010–0.0081)	0.0374(0.0076–0.0633)	0.7778(0.5493–0.9930)	-

### Model fitting and parameter uncertainty

A Bayesian frame work was used to fit the models to the calf data in R using the FME package [36] based on the procedure by [37] 2008) that implements the delayed rejection and adaptive metropolis algorithm simulated using the Markov Chain Monte Carlo (MCMC) method. Prior estimates of the model parameters were taken from our previous study and literature (See Table 2 for the cell compartmental model). For each parameter, a uniform prior was used with the lower and upper values taken as the parameter bounds and the average/median as the parameter estimate. In the fitting, we assumed that the observations have identically and independently distributed with additive Gaussian noise with unknown variance. Therefore, given data/observations ***D***, and a nonlinear model ***M***, (***M*** = *f*(***x***, ***θ***)), the data can be explained as ***D*** = *f*(***x***, ***θ***) + *error*, where *error* = *N*(0, *δ* ^2^) is the Gaussian noise. ***θ*** Are the model parameters to be estimated and ***x*** are the model time dependent variables. Applying the Bayesian probability, the model parameters can be drawn from the posterior distribution $P\left(D\;/M,\;\delta^{2}\right)=\frac{1}{P\left(D\right)}*\;P\left(M\;/D\right)P\left(M\right)$, where *P*(*M*) is the model prior, *P*(*D*) is the model evidence which in practice amounts to a normalising constant and *P*(*M* /*D*) is the model likelihood. Assuming uniform priors we estimated model parameters from posterior distributions generated using the MCMC method and a Gaussian likelihood function, *L*(*M*, *D*), therefore

$$L\left(M,D\right)\propto \prod_{i=1}^{n}\text{exp}(-{\left(D_{i}-M_{i}\right)}^{2}/2\delta^{2})=\prod_{i=1}^{n}\left[\text{exp}\left(\frac{-{\left(D_{IFN_{i}}-IFN_{i}\right)}^{2}}{2\delta_{1}^{2}}\right)\text{exp}\left(\frac{-{\left(D_{IL10_{i}}-IL10_{i}\right)}^{2}}{2\delta_{2}^{2}}\right)\text{exp}\left(\frac{-{\left(D_{IL4_{i}}-IL4_{i}\right)}^{2}}{2\delta_{3}^{2}}\right)\text{exp}\left(\frac{-{\left(D_{CFU_{i}}-CFU_{i}\right)}^{2}}{2\delta_{4}^{2}}\right)\right]$$ (3)

Dropping the constant terms we minimised the negative log likelihood, therefore

$$-\text{In}\;L\left(M,D\right)\propto \sum_{i=1}^{n}\frac{{\left(D_{IFN_{i}}-IFN_{i}\right)}^{2}}{2\delta_{1}^{2}}+\frac{{\left(D_{IL10_{i}}-IL10_{i}\right)}^{2}}{2\delta_{2}^{2}}+\frac{{\left(D_{IL4_{i}}-IL4_{i}\right)}^{2}}{2\delta_{3}^{2}}+\frac{{\left(D_{IL4_{i}}-IL4_{i}\right)}^{2}}{2\delta_{3}^{2}}.$$

This likelihood function was used in estimating parameters for the model in the two different modelling approaches, where *D*_*IFN*_, *D*_*IL*4_, *D*_*IL*10_, and *D*_*CFU*_ represent data experimental variables IFN, IL4, IL10 and CFU used in the first modelling approach, respectively. In the second approach, IFN and IL4 are represented by Th1 and Th2 variables, respectively. The data was normalised across all animals by dividing by the largest observed value of each data variable across all data sets, respectively.

### Parameters estimating, uncertainty and model selection

Uncertainty of the estimated parameters was evaluated by computing the 68% and 95% credible intervals (CIs). The CIs were evaluated by randomly sampling from the posterior distribution of each estimated parameter from the generated MCMC chain of 100000 runs. The MCMC chains were judged to have converged by visual examination and using the Coda convergence diagnostic tool in R. We also, carried further model parameter sensitivity analysis to show how model initial conditions affect model fitting (S3 Fig) and output variables (S4 Fig), and how multivariate variations of model parameters alter model predictions (see the supplementary file, S5 and S6 Figs). All models were fitted using the initial conditions (*M*_*ϕ*_(0) = 500.0, *I*_*m*_(0) = 0.0, *B*(0) = 1.0, *Th*_*o*_(0) = 0.1), while for the variables Th1, Th2, IL10 and CFUs, the first observed data values were used as the initial conditions. *I*_*m*_ was set to zero with the assumption that no shedding is observed therefore there are no productively infected macrophages (infected macrophages that are bursting and generating free bacteria). However, in S3 Fig we estimate the number of uninfected and infected macrophages, free bacteria, and naïve T cells for each animal, to understand how this potentially alters the predictions.

### Model selection

We used the Akaike Information Criterion (AIC) to compare model and select model mechanistic terms that best explain the experimental data, AIC = 2*log(Likelihood)+2*K. Where the likelihood is the probability of observing the data using the estimated parameters and **K** is the number of free parameters in the fitted model. AIC values of the models identified to best explain the data using each modelling frame work are given in Table 4. Also, the model fits were examined visually to aide model comparison and selection between models with complex and simple mechanistic structures when the same number of parameters was fitted (see S1 Fig). Models with a relatively high AIC value but with a less complex structure were given high precedence in model selection.

**Table 4:** Comparing models against calf experimental data.

Model	Calf data and AIC values			Model	Calf data and AIC values		
**Cell model**	Calf 1	Calf 2	Calf 3	**Cytokine model**	Calf 1	Calf 2	Calf 3
**Model 1**	18.1	21.4	24.1	**Model 1**	20.6	24.8	25.7
**Model 2**	19.3	20.5	19.2	**Model 2**	21.8	19.7	22.3
**Model 3**	22.2	21.9	18.7	**Model 3**	22.3	21.6	21.1

AIC values for models that were selected to explain the cytokine interactions in the 3 calves using the cytokine signalling model and cell compartmental model.

### Model simulations: Exploring the potential roles for IL-10

To investigate the different possible roles of IL-10 in MAP infection we used the cell compartmental model. This model was favoured because of its more realistic biological assumptions. In these simulations, we assumed that IL-10 can function in different capacities (or can have different roles). We assumed that IL-10 can (i) inhibit Th1 differentiation or proliferation, (ii) enhance Th1 differentiation or proliferation, (iii) can work in synergy with Th2 cells/response to inhibit Th1 differentiation or proliferation. IL-10 and Th2 synergy is assumed to take place in two different ways, (a) IL-10 can enhance Th2 differentiation (proliferation) while Th2 response suppress Th1 responses (differentiation and proliferation) and (b) IL-10 inhibit Th1 differentiation only while Th2 response suppress Th1 proliferation. Table 5 lists the possible functions of IL-10 tested and the mechanistic functions used to simulate the corresponding effects.

**Table 5:** Terms used for simulating IL-10 functions.

**IL-10 role assumed**	**Term used**
Th1 differentiation inhibition	*θ*_1_ *I*_*m*_ *Th*_0_/(1 + *a*_1_ *IL*_10_)
Th1 proliferation inhibition	*p*_1_ *I*_*m*_/((*I*_*m*_ + *M*)(1 + *a*_1_ *IL*_10_))
Th2 differentiation enhancing	*θ*_2_ *BTh*_0_ *IL*_10_
Th2 proliferation enhancing	*p*_2_ *BTh*_2_ *IL*_10_/(*B* + *M*)
**IL-10 role assumed in IL-10/Th2 synergy**	**Term used**
Th2 inhibit Th1 differention while IL-10 enhance Th2 differentiation	*θ*_1_ *I*_*m*_ *Th*_0_/(1 + *h*_1_ *Th*_2_) or *p*_2_ *BTh*_2_ *IL*_10_/(*B* + *M*)
IL-10 inhibit Th1 differentiation while Th2 suppress Th1 proliferation.	*θ*_1_ *I*_*m*_ *Th*_0_/(1 + *a*_1_ *IL*_10_) and *p*_2_ *I*_*m*_ *Th*_1_/((*M* + *I*_*m*_)(1 + *h*_2_ *Th*_2_))

Mechanistic terms used in simulating different immune regulatory functions of IL10 in MAP infection using then cell compartmental model.

## Results

### Comparing the experimental data to a Cytokine signaling model

**Fig 2:**
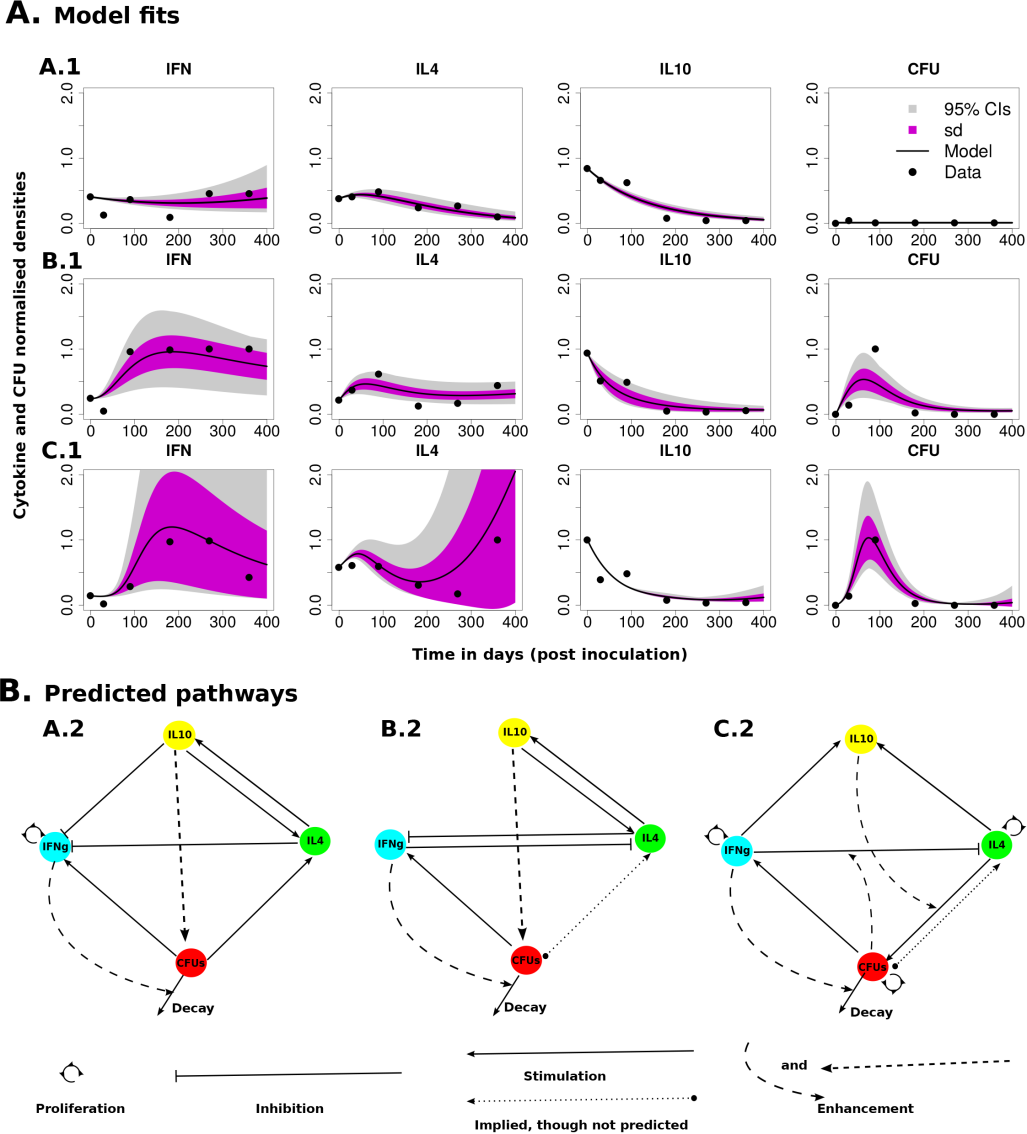
Model fitting and predicted immune interaction pathways. **A**) Shows how the predicted pathways represented by interaction maps/diagrams in **B** reproduce the experimental data. Best fits for calves A (A.1), B (B.1) and C (C.1) were obtained with models with immune interactions represented by signaling pathways A.2, B.2 and C.2 in **B**, respectively. The models used to predict these signaling pathways/maps are given in the supporting information file S1 Text, systems of equations (S1), (S2), and (S3), respectively. The first observed experimental values of each variable were used as the initial condition. The estimated parameters are given in Table 1 and they show the importance of these interactions. The magnitude of the estimated parameter indicates the strength of the interaction, while the 95%CIs show the uncertainty in the parameter estimates. The shaded regions represent the uncertainty in the model simulation results. **B**) Show the predicted biological interactions between IL-10, IFN-*γ*, and IL-4 that explain the immune kinetics and the CFU shedding dynamics of all the calves. Arrowed lines represent stimulated, dashed arrowed lines represent an enhancement of an effect, lines with a flat end represent the inhibition/suppression, and arrowed semi-circles represent proliferation/expansion. The dotted arrowed lines that start with a solid circle represent an indirect and implied stimulation that was predicted not to be essential to explain the data for some animals included to indicate that an infection is required for the initial cytokine stimulation but may not be important for their continued expression.

We used a cytokine signaling model to explain the interactions between the measured variables to understand the role of IL-10 in the regulation of Th1 (IFN-*γ*) and Th2 (IL-4) responses and how these responses correlate with CFU shedding. How these models explain the experimental infection data is shown in Fig 2A - A.1 (Calf A); B.1 (Calf B); and C.1 (Calf C). The models (system of equations) that corresponds to these fits are given by system of equations (S1), (S2), and (S3) (given in the supporting information file, S1 Text), respectively. The predicted immune interactions between IL-10, Th1 (IFN-*γ*), Th2 (IL-4) and B (CFU) are best explained by the interaction pathways/maps shown in Fig 2B (pathways A.2, B.2, and C.2 correspond to fitted patterns A.1, B.1 and C.1 in Fig 2A, respectively).

Using the cytokine signalling model, we predicted IL-10 to play potentially three different roles, (i) the inhibition/suppression of the Th1 (IFN-*γ*) response (Fig 2B: A.2 and B.2), (ii) enhancing disease progression in synergy with the Th2 (IL-4) response in all calves, and (iii) stimulate the expression of the IL-4 (Th2 response) (Calf A and B). Also, for all calves, we predicted the involvement of the Th2 (IL-4) response in the induction of IL-10 and the stimulation of IL-10 by Th1 (IFN-*γ*) response in Calf C which is supported by the study [38]. However other studies proved otherwise [39, 40]. The study of [41] showed that early immunologic events that stimulate Th2 cell differentiation require the role of IL-10. Induction of Th2 cell differentiation can be achieved through inhibition of IL-12 production from dendritic cells (DC) which leads to reduced IFN-*γ* (Th1 response) expression and hence subsequent development of a Th2 response [41, 42]. Apart from the fact that Th2 cells can secret IL-10, there is support from literature on the possibility of costimulation and synergy of IL-10 and IL-4 [43, 44]. Our model predicts synergy between IL-10 and IL-4 (Th2) responses may possibly enhance MAP infection, however, to the best of our knowledge we are not aware of any experimental evidence from MAP studies that support this theory and prediction. Also, there is evidence that IL-10 and IL-4 can act synergistically in suppressing *in-vitro* inflammation generated by Th1 responses [43, 45] and suppression of the protective Th1 response will favour MAP infection. In contrast, there are other studies that have suggested that IL-4 and IL-10 may be antagonistic [46] since IL-10 can play a dual regulatory role on both Th2 and Th1 responses. There is also an accumulating belief that the role of IL-10 could be key in developing MAP vaccines through immunoneutralisation of IL-10 to enhance IFN-*γ* production. Also, high IFN-*γ* expression observed in Fig 2A: A.1 explains low CFU shedding. Low or no CFU shedding implies an infection that has not progressed, which is normally a result of a strong sustained IFN-*γ* (Th1) expression. IL-4 and IL-10 are predicted to suppress IFN-*γ* expression, however, since their expressions are going down, IFN-*γ* will remain relatively highly expressed.

Fig 2B: B.2 and C.2 (predicted interaction pathways for Calf B and C) suggest inhibition of IL-4 (Th2) responses by IFN-*γ* (Th1) responses, while in calf A the reverse is predicted—inhibition of Th1 by the Th2 response is required to explain the data. The development of both IFN-*γ* (Th1) and IL-4 (Th2) mediated immune responses are predicted to be orchestrated by the MAP infection through differentiation and proliferation, however, in the development of the Th2 response, IL-10 is predicted to play a supportive role as opposed to the suppressive role on the IL-4 (Th1) response. A Th2 response is predicted to also have a protective role in Calf A, while in calves B and C the Th1 responses are predicted to have a protective role. Another prediction we found fascinating is on the potential role played by the MAP infection burden. In Calf C, we predicted that MAP bacterial density (potentially both extracellular and intracellular bacteria, however, most likely intracellular bacteria) can potentially act in synergy with the Th1 responses to inhibit Th2 responses. This observation seems to suggest that the burden of intracellular bacteria may influence the switching in the Th1/Th2 dominance. Infected macrophages or intracellular pathogens are believed to skew the differentiation of Th0 cells into Th1 cells through secreting IFN-*γ* and IL-12. Furthermore, IFN-*γ* can activate resting macrophages to kill intracellular bacteria.

### Comparing the experimental data to the cell compartmental model

**Fig 3:**
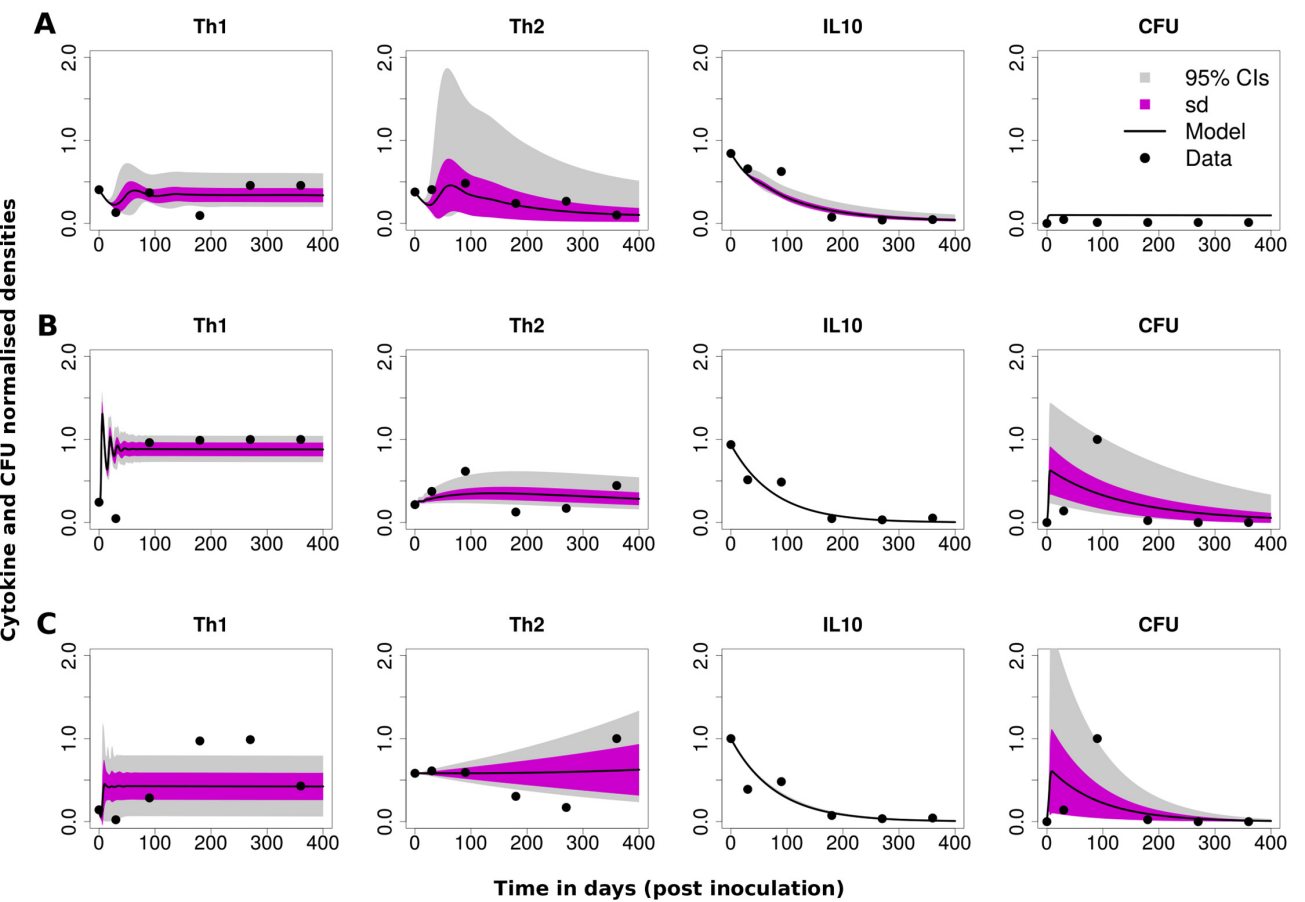
Cell compartmental model data fitting. The best fit for Calf A was obtained with a model that assumed IL-10 inhibits Th1 response expansion. Calf B data was best explained by a model that assumed IL-10 enhances Th2 proliferation with Th1 and Th2 differentiation cross inhibition. To obtain the best fit for Calf C, a model that assumed Th1 inhibition by IL-10 and Th2 inhibition by Th1 was used. The estimated parameters are given in Table 3 and the parameters that were fixed during model fitting are given in Table 2.

The data can also be explained using the cell compartmental model as illustrated in Fig 3. The patterns observed in the data can be explained using a combination of immune response, infection and shedding parameters (see Table 3). The parameters *k*_*i*_, *k*_*b*_, *λ*_1_, and *δ*_*m*_ were predicted to be essential to explain data for all calves. Also the parameters *θ*_2_, *u*_*cf*_, *p*_2_, *δ*_*Treg*_, *δ*_*B*_, were predicted to be either relevant or not relevant between the calves. In Table 3, a dash (-) implies that a given parameter was not essential to explain the data kinetics of a given calf. The magnitude of the parameter quantifies the importance of the parameter or the influence of the associated biological mechanism in the dynamics, hence predicting different and similar biological processes between the animals. These predicted parameters can be grouped into 3 categories (i) the infection parameters (*k*_*i*_, *k*_*b*_), (ii) immune response parameters (*δ*_*m*_, *δ*_*B*_, *δ*_*Treg*_, *θ*_2_, *u*_1_, *u*_2_, *p*_2_) and (iii) the MAP bacteria shedding parameters (*λ*_1_, *u*_*cf*_). In our model fitting, we assumed that IL-10 is produced by Treg cells indirectly through the density of infected macrophages (see S2 Table for more details on how this term was selected) [24, 29], that is IL-10 is assumed to be generated in proportion to the population of infected macrophages using either a mass action term (or density dependent term) between Th0 cells and infected macrophages. Through this analysis we identified that different roles of IL-10 can generate different immune responses. Calf A, was best explained with a model with IL-10 inhibiting Th1 cell differentiation, Calf B data was best matched when IL-10 promoted Th2 cell proliferation and in Calf C, when IL10 enhanced Th2 cell differentiation. We also tested these models against all the calves (see Table 4) and in some cases we could not find statistical evidence to select one model over the others, however, through visiual exmanination of the fits we could clearly discriminate the best models (see S1 Fig for an illustration of the visiual examination). Also, we observe (S3 Fig) similar predictions when we fit for the initial populations of uninfected and infected macrophages, free bacteria and naïve T cells. However, fixing the initial populations of these cells at the same values during fitting gives a better platform for comparing mechanisms between the animals, since altering initial conditions affects the transient kinetics of the disease but not its long term dynamics (see S4 Fig).

To explain bacteria shedding (CFUs data), we selected the use of the term, *λ*_1_ *N*_*o*_ *I*_*m*_, which explains shedding as a function of infected macrophages–the assumption that infected macrophage migrate into the intestinal lumen and thereby increase CFU in the feces. This selection is based on a plausible biological explanation that supports the theory that MAP require a mechanism or a vehicle for transportation in a similar fashion to the process that facilitates infection, where entry and passage of bacteria is via M-cells and enterocytes [47, 48]. However, there is a gap in current knowledge about the exact mechanism on how bacteria are leaked into the lumen from infected tissue and lesions. Our predictions and model assumptions are sensible since it seems implausible that free bacteria will migrate on their own into the gut unless the epithelial cell lining is severely compromised. Also, there is evidence that gastrointestinal epithelium deploys several mechanisms to fight microbial intruders and one potential mechanism is the expulsion of infected cells [49]. In Salmonella infection, it was demonstrated that epithelial cells laden with bacteria in the cytosol are extruded out releasing invasion-primed and competent Salmonella into the lumen [50].

In Fig 3A (Calf A), IL-10 is predicted to inhibit the differentiation of Th0 cells into Th1 effectors. However, to explain data of Calf B, we introduced a proliferation term, ($p_{2}T_{h_{2}}I_{L_{10}}\frac{B}{B+M_{ST}}$). This suggests that IL-10 could be involved in enhancing the expression of a Th2 (IL-4) response or that the expression of Th2 is linked with the expressed levels of IL-10. This identifies a different role/function for IL-10 compared to its predicted role in Calf A. Overall, there is an initial high expression of IL-10 which declines over time for the three calves. The IL-10 expression pattern is matched initially by a relatively low Th1 expression that then increases as the IL-10 expression declines. These patterns are associated with early substantial MAP shedding and then CFU levels are pushed down as Th1 expression increases, suggesting a Th1 protective role. The reduced level of IL-10 and CFU shedding are clearly explained by our model through the reduced levels of infected macrophages that are observed as Th1 expression increases. Reduced levels of infected macrophages will lead to reduced generation of IL-10 (supposedly by Treg cells). This observation is supported by several experimental studies that showed that monocytes and macrophages can be a significant source of IL-10 in response to mycobacterial antigens. This is also true for Treg cells. There is growing evidence that the IL-10 observed in MAP infected cattle is produced by CD4+CD25+ cells (Treg cells) [29]. We predicted that the increased Th2 response observed for Calf B (Fig 3B) after about 300 days, is probably maintained through proliferation. However, the role of differentiation, parameter *θ*_2_, is required for the early generation of Th2 cells and in the conversion of Th0 cells into Th2 effectors.

To describe Calf C data (Fig 3C), Th2 differentiation term was predicted to be important. Also, we predicted that Th1/Th2 differentiation cross inhibition is required to explain the decline in Th1 expression that is observed as Th2 expression increases. IL-10 is predicted to have a dampening effect on Th1 cells, however IL-10 expression can not be sustained as the level of infected macrophages and free bacteria are kept low by a combined Th1 and Th2 response. We also observe just like in the other calves that there is substantial CFU shedding in the first 100 days, which is associated with increased infected macrophages and bacteria density within the same time frame. This shedding is observed to decline with increased Th1 expression as well as the IL-10 expression declines.

### Exploring potential roles/functions of IL-10 in MAP infection

#### Inhibition of Th1 by IL-10 enhance MAP infection

**Fig 4:**
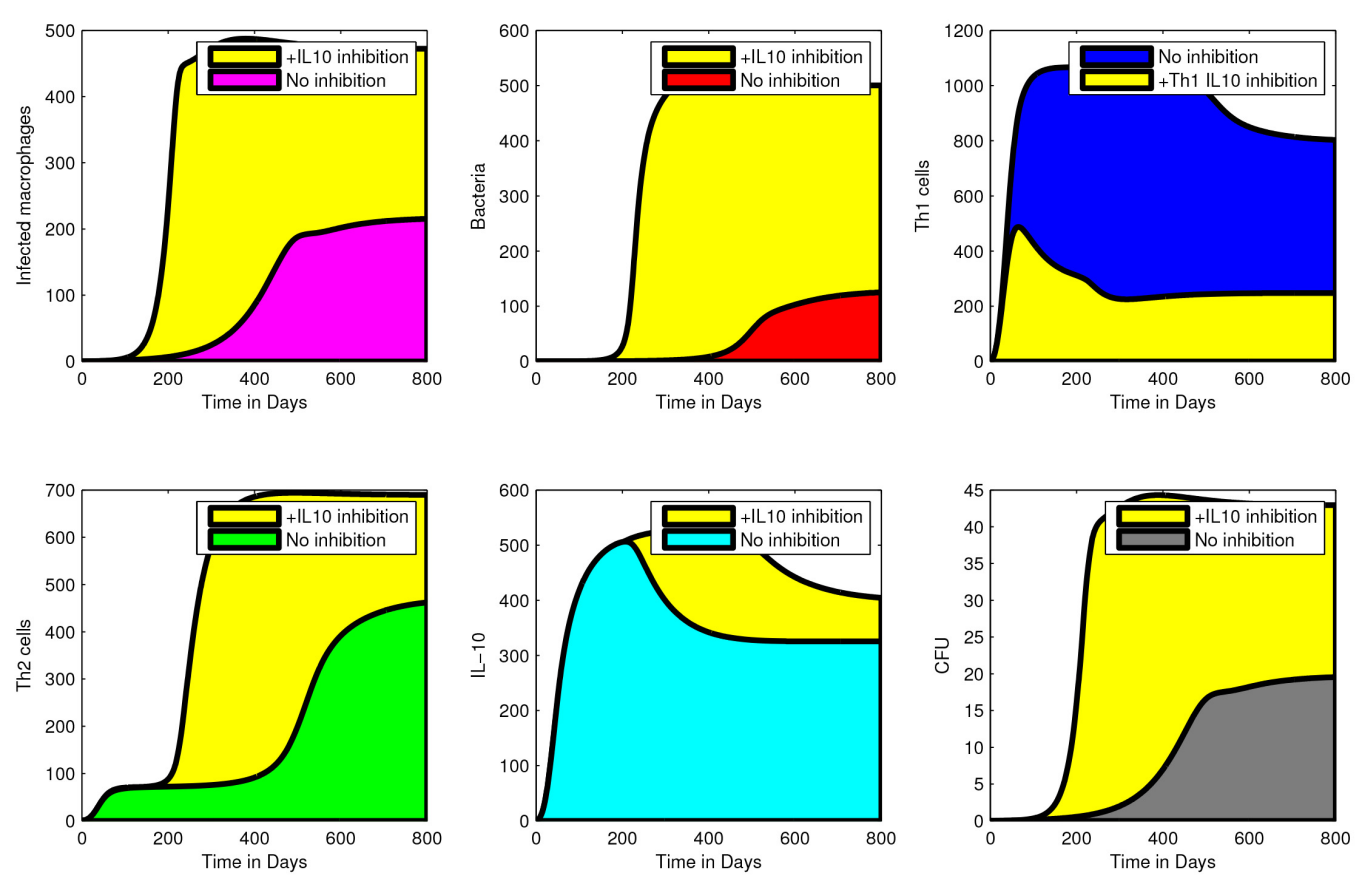
An illustration of the effect of IL-10 Th1 inhibition. Simulations showing the effect of IL10 inhibition on Th1 cells and how this translates to other cell populations and CFU shedding. Increasing IL-10 inhibition on Th1 cells results in reduced Th1 expression. Suppression of Th1 response is followed by a high density of infected macrophages, extracellular bacteria, and Th2 expression. These changes explain the increased MAP (CFU) shedding. Simulations were generated using the cell compartmental model with the assumption that IL-10 inhibits Th1. IL-10 inhibition is modelled by increasing the inhibition parameter, *a*_1_ from 0 (no inhibition) to 0.005 (+IL-10 inhibition) while the rest of the model parameters are kept at the baseline values given in Table 2. The yellow shading shows the effects (population increase or reduction) as a result of IL-10 inhibition. The shading of cell populations before IL-10 inhibition are represented by the colours: grey-CFUs, blue-Th1 cells, red-bacteria, cyan-IL-10, green-Th2 cells, pink-infected macrophages.

Fig 4 shows the effect of IL-10 inhibition on Th1 responses (assuming that IL-10 is expressed in MAP infections and its role is to suppress Th1 (IFN-*γ*) expression). Suppression of Th1 by IL-10 is shown to be associated with increased MAP infection. When IL-10 inhibition is increased the density of infected macrophages, free bacteria, Th2 expression and CFU shedding are increased while Th1 expression diminishes. This result also shows that high expression of IL-10 is matched with a corresponding expression of the Th1 response and the decline in the Th1 expression is followed by a drop in IL-10 expression. This observation from our model is consistent with observations from experimental studies [7, 20], which showed high expression of both IL-10 and IFN-*γ*. Since the Th1 response is protective, its high expression will be matched by less CFU shedding. If the role of IL-10 is to suppress or inhibit IFN-*γ* (Th1), then when Th1 expression decreases, the bacterial load will increase with increasing Th2 (which is assumed not to be protective). Increasing bacteria results in more infected macrophages hence increased CFU shedding.

#### IL-10- Th2 (IL-4) synergy and inhibition of Th1 (IFN-*γ*) favours MAP infection

**Fig 5:**
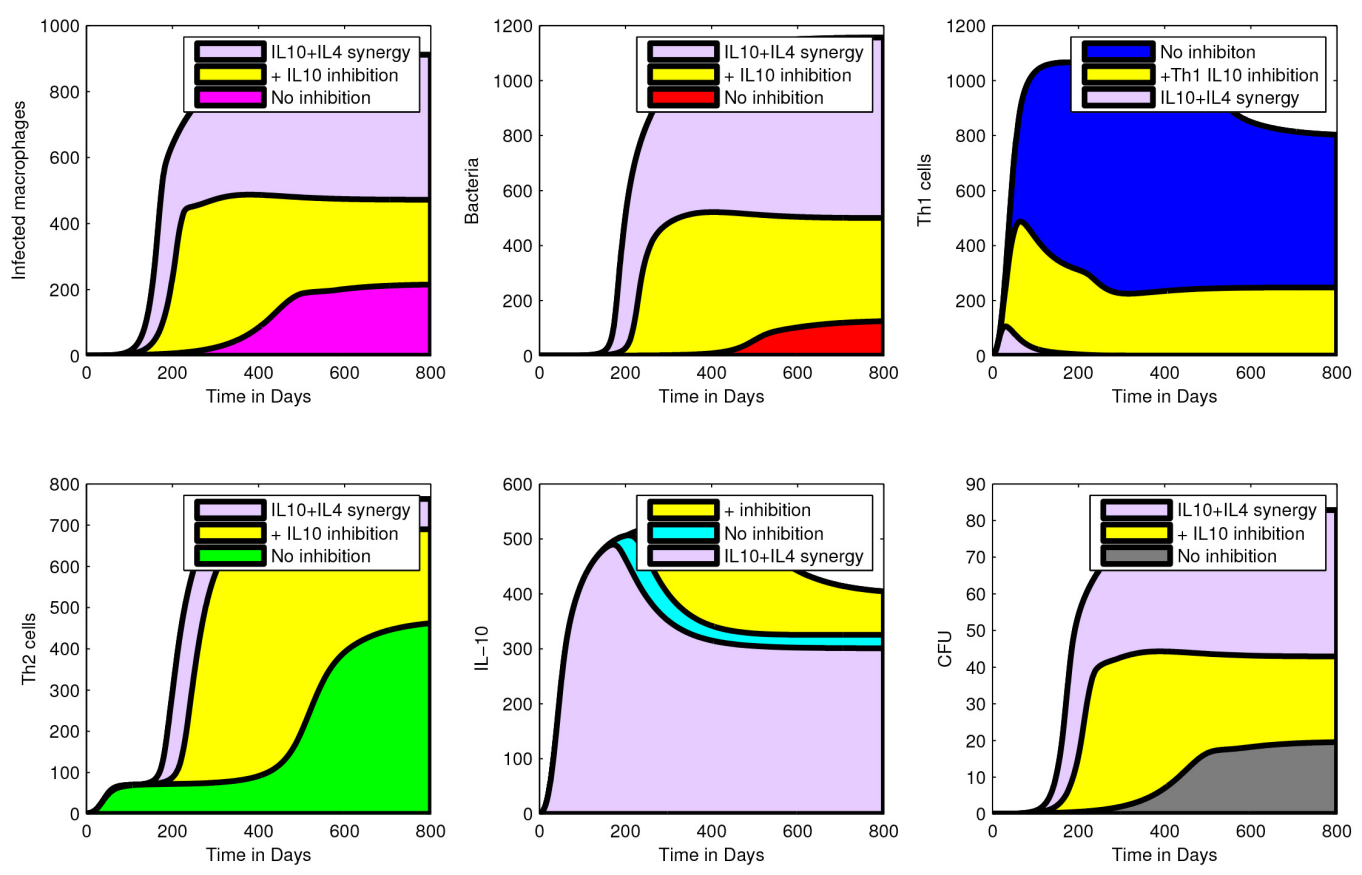
Effect of IL10—Th2 (IL4) synergy inhibition on Th1 cells. Simulation showing increased suppression of Th1 cells when there is IL-10—Th2 (IL4) synergy. Suppression of the Th1 response accompanied by loss of protection, increased disease progression and CFU shedding. Simulation of IL-10—Th2 (IL4) synergy were generated by a model with the assumption that IL-10 and IL-4 (Th2) interact synergistically to inhibit the expression of the Th1 responses. IL-10—Th2 (IL-4) synergy was modelled using the term, $\left(\frac{1}{1+a_{1}I_{L_{10}}T_{h_{2}}}\right)$, with *a*_1_ = 0.005 while the rest of the model parameters are kept at their baseline values given in Table 2. The shading of cell populations before IL-10 inhibition are represented by the colours: grey-CFUs, blue-Th1 cells, red-bacteria, cyan-IL10, green-Th2 cells, pink-infected macrophages. The yellow shading represents the effect of IL-10 inhibition only, while the violet colour represents the effects of IL10 and IL4 synergy.

To investigate the effect of IL-10-Th2 (IL4) synergy in MAP infection, we assumed two different roles for IL-10. First, we assumed that IL-10 can suppress Th1 expansion while at the same time the expressed Th2 (IL-4) will suppress Th1 expression (*see* the supplementary information and Table 5 on how IL-10—Th2 (IL4) synergy was modelled). Using this assumption, we observed increased cell populations (shown in Fig 5) except for the population of Th1 cells. These simulations show the difference in Th1 suppression achieved when only IL-10 inhibition is assumed (no synergy) and when there is IL-10—Th2 (IL4) synergy. Apart from showing increased MAP infection, simulations also predict increased bacterial shedding associated with IL-10—Th2 (IL4) synergy. Also, Fig 5 show that as the level of the expressed Th1 cells decline, IL-10 expression also declines, however, this declining effect is only translated/transferred to a slow steady increase in the population of infected macrophages and free bacteria. Once Th1 expression is depleted, the population of infected macrophages and free bacteria will increase even when the Th2 (which is not protective) expression is increased. Increased population of infected macrophages will lead to increased CFU shedding, while free bacteria will skew Th0 cell differention towards Th2, hence a dominant Th2 response. Once Th1 expression is low, infected macrophages will accumulate. This will not help differention selection to favour Th1 expansion because of the IL-10—Th2 (IL4) synergy. Second, we assumed that IL-10 enhances Th2 cell proliferation (IL-4 expression) while the Th2 response inhibits Th1 differentiation. S2 Fig shows that this assumption will achieve qualitatively simular results to the results of the first assumption. However, the mechanism through which these results are achieved are different. In this case, IL-10 does not directly inhibit Th1 cells, but does so through enhancing Th2 expression, which in turn suppresses the Th1 response.

## Discussion

The role of IL-10 in MAP infection in Johne’s disease (JD) has recently garnered interest and has become a subject of research focus. There is accumulating evidence that IL-10 may play a critical role in modulating the adaptive immune responses hence influencing disease pathology. Also, there is a belief that immunoneutralisation of IL-10 can unlock several cues for the design of new MAP vaccines that can enhance IFN-*γ* production. There are studies that have showed that IL-10 suppress IFN-*γ* responses, this in turn favours disease pathology [13, 16]. However, other studies suggested both early expression of IL-10 and IFN-*γ* to be associated with disease protection [7, 20].

We applied mathematical modeling as a gateway into gaining insights and further understanding of the roles played by IL-10 in MAP infection. We developed and employed two different mathematical models to explain the MAP experimental infection data (IL-10, IFN-*γ* (Th1), IL-4 (Th2), and CFU (bacteria)) from calves that were followed over a period of 360 days in the study [15]. We confronted our models with the data (see Figs 2 and 3) and we showed several ways the Th1 and Th2 responses interact, the role of IL-10 in each animals and how the immune response influences the MAP bacteria shed in feces. The cell compartmental model identified three sets of parameters that are important to explain the data for all the calves: (i) the infection parameters (*k*_*i*_ and *k*_*b*_), (ii) the immune response parameters (*δ*_*m*_, *δ*_*B*_, *δ*_*Treg*_, *θ*_2_, *u*_1_, *u*_2_, *p*_2_) and (iii) the MAP shedding parameters. These parameters suggest that different rates of macrophages infection (*k*_*i*_) and bursting (*k*_*b*_) are important for infection establishment and their magnitutdes correlate with disease progression. Also, immune response mechanisms (associated with Th1, Th2 and IL10), that is Th1 effector function, Th1 inhibition by the Th2 response, suppression of Th1 by IL10 and the role of IL-10 in mediating Th1 suppression by the Th2 response contributes to the different immune dynamics and disease trajectories that are observed.

Our cytokine signalling model results suggest different mechanisms through which different Th1/Th2 pathways can be regulated by IL-10 and illustrates how these mechanisms potentially influence MAP shedding. IL-10 is predicted to inhibit IFN-*γ* (Th1 response) expansion (in Calf A and Calf C), enhance IL-4 stimulation (Th2 response) (calf B), and inhibit expansion of Th1 response while IFN-*γ* inhibit IL-4 (Th2) expansion (Calf C). Our model predictions are consistent with data for all the calves, that is, high levels of IFN-*γ* (Th1 response) is matched with no MAP shedding and low IL-10. While substantial shedding is correlated with high IL-10. Also, our models could explain the declining effect of IL-10 on (i) IFN-*γ* (Th1) inhibition as the IL-10 expression plummets hence the observed increased IFN-*γ* (Th1 response) expression, and (ii) on inducing IL-4 (Th2) expression as IL-10 expression falls and therefore the role of IL-4 expansion cause prolonged high stimulated levels of IL-4 (Th2 responses) in calves B and C (as shown in Fig 2A and 2B). In all the calves substantial shedding that correlates with early high IL-10 and Th2 expression is observed, and no shedding is observed when IFN-*γ* is high and when IL-10 is low. Our model reproduces data patterns and hence predicts potential biological mechanisms through which the observations can be generated. We show that high shedding is associated with high intracellular bacterial burden (population of infected macrophages) and a relatively low Th1 response (*see* Figs 3, 4 and 5). The model also shows that high Th1 expression infers protection while a Th2 response is associated with increased bacterial burden hence rapid disease progression and increased bacterial shedding.

The results predicted using the cell compartmental model were matched with the predicted immune mechanisms identified using the cytokine signaling model. We eliminated feasible cytokine signaling biological interactions between IL-4, IL-10, and IFN-*γ* that could not help improve the model in describing the cytokine and CFU data. We identified three different biological pathways/maps and these predict the immune mechanisms and biological interactions that were stimulated in each calf. This analysis revealed further how IL-10 interacted with IL-4, and IFN-*γ*, and how the signaling between these three cytokines affected and contributed to the observed CFU shedding. The predicted signaling pathways illustrate that IL-4 (Th2 response) expression to be linked with increased MAP shedding, hence disease progression. We predicted similar results using the cell compartmental model. This observation is supported by the current understanding in MAP disease that a Th2 response is non-protective and the dominance of Th2 response occurs with concomitant increased bacteria shedding. We also predicted IL-10 to be synergistically linked to the role of IL-4 in MAP infection and that their combined role potentially enhances disease pathology (similar predictions were predicted using the cell compartmental model). Synergy between IL-10 and IL-4 is still to be experimentally illustrated in JD. Whether the IL-10-Th2 (IL-4) synergistic relationship is pro-disease as predicted by our models (Figs 4 and 5 and S2 Fig) or not, still remains an open question to be addressed. However, there are several other experimental studies of other infections that support our predictions [43–45].

The role of IL-10 predicted with the cytokine signaling model is similar to the prediction of the cell model model that showed that IL-10 can enhance the Th2 response (which does not offer disease protection). Or it can suppress the Th1 response which is counter effective to disease protection. Suppression of the Th1 response by IL-10 could also be important for balancing pro-inflammatory responses resulting in quiescence or latency of the immune response. Whichever role assumed by IL-10 will result in enhanced Th2 expression. The humoral response (Th2 response), was shown in the study of [51] to enhance bacteria opsonisation by macrophages, and it is well known that mycobacterial pathogens flourish on replicating in the macrophage intracellular environment. An IL-10 suppression of Th1 cells is predicted in Calf A and B. Surprisingly [38], Th1 (IFN-*γ*) response is predicted to enhance (to promote) IL-10 expression in Calf C. Biological mechanisms that regulate Th1 cells to directly or indirectly secrete IL-10 are not clearly known but several studies reviewed in Cope et al [52] suggest and illustrate the existence of such molecular mechanisms that cause a switch from IFN-*γ* production to IL-10 by Th1 cells. Expression of IL-4 is predicted to favour the expression/stimulation of IL-10. We also, predicted IFN-*γ* to inhibit IL-4 (*see* Fig 2B: B.2 (Calf B) and C.2 (Calf C)), while in calf A IL-4 is predicted to inhibit Th1 (IFN-*γ*) response. These results suggest that even though Th1/Th2 cross inhibition is a known biological process, it may be significantly skewed in one way or the other in different animals, this is also true with the role of IL-10 between animals. Heterogeneity in the Th1, Th2 and IL-10 functions could be central to different immune-pathologies that are observed in MAP infected animals.

Our model analysis is limited in providing predictions that show the benefit of IL-10 suppression of Th1 systemic inflammation or providing predictions of the associated implications. There are several infections that can be successfully treated with inflammation therapy. MAP infection studies are still needed to clearly enumerate quantitatively the trade-off between the benefits of Th1 responses accompanied by the inflammation and the cost (potential disease pathology) of IL-10 Th1 suppression and to provide clear interpretation whether at the animal level IL-10 indeed confers protection while at the site of infection favours pathology. In order to keep our cell compartmental model simple, we did not incorporate explicitly the role of Treg cells. Availability of experimental data that show the time kinetics of Treg cells and how it correlates with other immune mechanisms will make it more sensible to improve our model and make disease prediction more informative. We also predicted that bacterial burden can enhance Th1 suppression on Th2 responses. Another weakness of the detailed model is that we left out several Th1/Th2 biological processes such as proliferation, proliferation inhibition, effector function inhibition, T cell exhaustion and other cell lines such as Th17, Treg and their potential roles. In our previous study [26] we showed how several Th1/Th2 biological mechanism influence disease dynamics. The role of Tregs and Th17 cells are still to be investigated, however there are still limitations in the availability of quantitative data for these to be evaluated using mathematical models. The main shortfall of the cytokine signaling and CFU shedding models is that it simplifies the mechanistic interactions between IL-4, IL-10, and IFN-*γ* without giving exact details of the underlying biological pathways. This model makes an oversimplifying assumption that the excreted CFUs represent the within animal MAP bacteria, though we know that CFU shedding is a sign of disease progression. Therefore, the model best explains the interactions of the already expressed immune variables and the excreted CFUs. Use of cytokine time course expression data extracted over the course of MAP infection will make it possible to decipher these signaling pathways and how their cross talk skew specific adaptive responses in MAP infection. Differential expression of IFN-*γ* and T-bet transcription factor are believed to skew differentiation toward the Th1 lineage while IL-4 and GATA-3 favour Th2 selection lineage (reviewed in [53]).

Apart from IFN-*γ*, IL-4 and IL-10, there are several cytokines, such as IL-12, IL-2, IL-1, IL-18 and TNF-α that are involved in the regulation of immune responses to bacterial infections. IL-18 plays an important role in differentiation of naïve T cells to Th1 cells and act as amplifying signal for IFN-*γ* production [54]. In a mouse of *Mycobacterium tuberculosis* (Mtb) infection, mice deficient of receptor for IL-1 were found to be more susceptible to Mtb infection and exhibited outgrowth of bacteria, developed defective granulomas and had decreased IFN-*γ* production [55]. TNF-α is known to be important in granuloma formation and can induce killing of Mtb through the production of reactive oxygen species [56]. Also, TNF-α acts in synergy with IFN-*γ* and mediates Mtb killing through the production of reactive nitrogen intermediates [57, 58]. The signaling between these cytokines is still to be completely explained and it is our belief, that with the availability of experimental data were these cytokines are completely enumerated, our mathematical models will be key in predicting and inferring how they interact and to explain bacterial (such as MAP infection) infections. For instance, in this study, we did not capture the contribution of IL-12, which is another important cytokine for MAP infection. IL-12 induces IFN-*γ* production and is mostly produced by phagocytic cells in responses to bacteria, bacterial products, intracellular parasites and to some degree by B lymphocytes. Early in the infection, IL-12 induces IFN-production from NK and T cells, which contributes to phagocytic cell activation and inflammation (59, 60). IL-12 and IL-12-induced IFN-*γ* favor Th1 cell differentiation by priming CD4+ T cells for high IFN-gamma production and also contributes to IFN-*γ* optimal production to support the proliferation of differentiated Th1 cells in response to antigen [59]. The selection for early expressed immune responses depends on the balance between IL-12, which favors Th1 responses, and IL-4, which favors Th2 responses [59]. Thus, IL-12 represents a functional bridge between the early nonspecific innate resistance and the subsequent antigen-specific adaptive immunity [59, 60]. In the early stages of MAP infection, Th1 cytokines, such as IFN-*γ* (and IL-12),IL-2 and TNF-α, were shown to be expressed in infected animals [61, 62]. Production of IFN-*γ* and IL-2 was identified to be reduced in cows with clinical JD [61–63] whereas expression of a Th2 cytokine (IL-4) was elevated [64]. Th2 cytokine, IL-4, suppresses macrophage activation caused by IFN-*γ* [65] and also inhibit autophagy-mediated killing of intracellular mycobacteria [66], hence enhancing disease progression. As more data become available, our future modeling work will seek to explain MAP infection and address some of the pressing questions in JD. However, mathematical models only help us to understand the biological systems where measuring biological variables is limited, experimental resolution could be lacking and in predicting probable underlying biological mechanisms that explain the patterns observed in the data.

Based on our model predictions we recommend experiments that (i) will evaluate and quantify the implications of IL-10-Th2 (IL-4) synergy in MAP infections, (ii) will investigate how intracellular bacterial burden manipulates Th1 responses and evaluate how this translates to IL-4 and IL-10 expression. Mycobacterial infection are known to have the ability to somehow manipulate the immune surveillance and signaling pathways. And (iii) experiments to decipher the exact mechanism through which bacteria are leaked from lesions and infected tissue into the gut before shedding.

## Supporting Information

S1 Fig Model fitting visual examination. An illustration of model comparison and selection of models with close AIC values using visual examination or eyeballing. It is very clear that Model 1 fails to adequately explain all trends. However, the AIC values do no give enough information to select either Model 2 or Model 3. But by visually examining the fits, it becomes clear that Model 3 explains IL-4 and IFN-*γ* much better than Model 2 (**A** and **B**). These two models seem to explain IL-10 and CFU patterns in more or less a similar way (**C** and **D**).
(PDF)

S2 FigThe effects of IL-10-enhanced Th2 proliferation and inhibition of Th1 expression by Th2.Simulations showing the effect of IL10- Th2 (IL4) synergy through (i) IL-10 enhancing Th2 cell expansion and (ii) Th2 suppressing the Th1 differentiation. Enhanced Th1 suppression is observed between the achieved suppression level of Th1 by the Th2 response only and the suppression level achieved through IL-10 and Th2 (IL-4) synergy. IL-10- Th2 (IL4) synergy was modelled using the terms (i) $\left(\frac{\theta_{1}\delta_{m}I_{m}T_{h_{0}}}{1+b_{1}T_{h_{2}}}\right)$ (Th1 (IFN-*γ*) inhibition by Th2 (IL-4)) and (ii), $\alpha_{1}T_{h_{2}}I_{L_{10}}(\frac{B}{B+M_{S}})$ (Th2 proliferation), with *b*_1_ = 0.005 and *α*_1_ = 0.0002 while the rest of the model parameters are kept at the baseline values given in Table 2. The shading of cell populations before IL-10 inhibition are represented by the colours: grey-CFUs, blue-Th1 cells, red-bacteria, cyan-IL10, green-Th2 cells, pink-infected macrophages. The yellow shading represents the effect of IL-10 inhibition only, while the violet colour represents the effects of IL10 and Th2 synergy on the inhibition of Th1 expression.(PDF)

S3 FigCell model fits with initial fitting.We show how fitting initial conditions, together with parameters alter the model fits. There is no much variation in the fitted model trajectories between S3 Fig, however, different parameters are estimated. The estimated parameters and initial conditions are given and their uncertainties are shown by the 95% CIs given in brackets. The shaded regions in this figure show the uncertainty in the model predictions that correspond to the estimated parameters (without including the uncertainty in the initial conditions). **Calf A)** *k*_*i*_ = 0.0809(0.1466–0.0052), *k*_*b*_ = 0.00780(0.01411–0.00071), *λ*_1_ = 0.0123(0.0169–0.0103), *δ*_*m*_ = 0.0074(0.0107–0.0050), $\delta_{T_{reg}}$ = 0.1987(0.2909–0.1078), *θ*_2_ = 216.85(412.45–104.75), *δ*_*B*_ = 0.0803(0.0989–0.0496), *M*(0) = 0.0633(0.1296–0.0304), *I*_*m*_(0) = 0.4371(0.6397–0.2049), *B*(0) = 0.0234(0.0490–0.0012), *Th*_0_(0) = 68.67(98.25–29.54). **Calf B)** *k*_*i*_ = 0.5308(0.5682–0.3402), *k*_*b*_ = 0.0207(0.0229–0.0168), *λ*_1_ = 0.0962 (0.1056–0.0618), *δ*_*m*_ = 0.4359(0.5200–0.3384), *p*_2_ = 1.0306(1.1713–0.6776), *μ*_1_ = 0.1861(0.2129–0.1208), *δ*_B_ = 0.0367(0.0437–0.0251), *μ*_*CF*_ = 0.0115(0.0127–0.0072), *M*(0) = 47.82(50.75–39.36), *I*_*m*_(0) = 6.5560(8.0265–4.2926), *B*(0) = 0.13801(0.1677–0.0935), *Th*_0_(0) = 2.1774(2.3677–1.4161). **Calf C)** *k*_*b*_ = 0.0076(0.0109–0.0052), *λ*_1_ = 0.1290(0.1801–0.0844), *δ*_*B*_ = 0.0115(0.0154–0.0081), *a*_1_ = 0.0360(0.0674–0.0141), *a*_2_ = 0.8066(1.1799–0.4908), *μ*_1_ = 0.0359(0.0668–0.01139), *μ*_2_ = 1.2369e-05(1.9019e-05-8.1854e-06), *k*_*i*_ = 0.6753(0.9920–0.4471), *M*(0) = 329.43(545.35–183.93), *I*_*m*_(0) = 3.8573(8.4403–1.1546), *B*(0) = 0.5246(0.7719–0.3122), *Th*_0_(0) = 3.7191 (5.1772–2.6257).(PDF)

S4 Fig Cell model qualitative analysis of model parameters and initial conditions. In panels (**A**, **B** and **C**) Initial conditions for bacteria dose, naïve T cells and Th1 cells were varied to evaluate how the steady state solutions of the model change. In panels (D, **E** and **F**) the rate of macrophage infection (*k*_*i*_), Th1 effector function (*k*_*l*_), and the infected macrophage burst size (*N*_*o*_) were varied to show how changing these parameters alter the disease (CFU shedding) endpoints. Increasing the Th1 effector function leads to a no shedding stable state, while increasing infection of macrophages and the amount of bacteria released at bursting leads to a stable CFU shedding state. Also, increasing the size of Th1 and Th0 cells leads to a no CFU shedding state, while increasing initial bacteria size exposure increases chances of disease, hence a stable CFU shedding state.
(PDF)

S5 Fig Cell model parameter sensistivity analysis. The model represented by system of Eq 2 was used to illustrate the model output sensitivity to variation in several model parameters (*k*_*i*_, *k*_*l*_, *k*_*m*_, *k*_*b*_, *N*_*o*_, *δ*_*B*_, *λ*_1_, *μ*_1_, *μ*_2_, *μ*_*cf*_). The the uncertainty in model output variables is shown using grey (5^th^-95^th^ quantiles) and magenta (25^th^-75^th^ quantiles) coloured regions.
(PDF)

S6 Fig Cytokine model parameter sensistivity analysis. Model 3 (for calf C) was used to demonstrate the effects of varying several model parameters (*α*_1_, *α*_2_, *α*_3_, *α*_4_, *μ*_1_, *μ*_2_, *μ*_3_, *μ*_4_, *r*) to show the uncertainty in model output variability. The shaded regions show the 5^th^-95^th^ quantiles (grey) and (magenta) 25^th^-75^th^ quantiles, in the simulated trajectories.
(PDF)

S1 Table Selection of the shedding terms. AIC values used to select the mechanistic terms used to model MAP CFU shedding in the cell compartmental model. The term *λ*_1_ *I*_*m*_ was selected because it is believed that MAP bacteria can escape from the intestine into the gut via infected cells. Also, there is no strong statistical information to select one term over the other.
(DOCX)

S2 Table Selecting terms that are used to generate IL-10 in the models. Terms used to model the production of IL10 in the cell compartmental model and their corresponding AIC values. The term, *θ*_3_ *I*_*m*_ *Th*_0_, was selected to model IL10 because of its associated AIC value.
(DOCX)

S1 Text Signalling cytokine models. Mathematical models used to generate the predicted immune pathways that are shown in Fig 2B. S1 Text gives a brief explanation of the immune interactions that we predicted to be essential to explain the calf data and explains the simplifying assumptions of the empirical models.
(DOCX)
