# Supplementary figures and images for: Predicting the Role of IL-10 in the Regulation of the Adaptive Immune Responses in Mycobacterium avium Subsp. paratuberculosis Infections Using Mathematical Models
Source: PLoS One. 2015 Nov 30;10(11):e0141539. doi: 10.1371/journal.pone.0141539 (PMC4664406; doi:10.1371/journal.pone.0141539)

**A**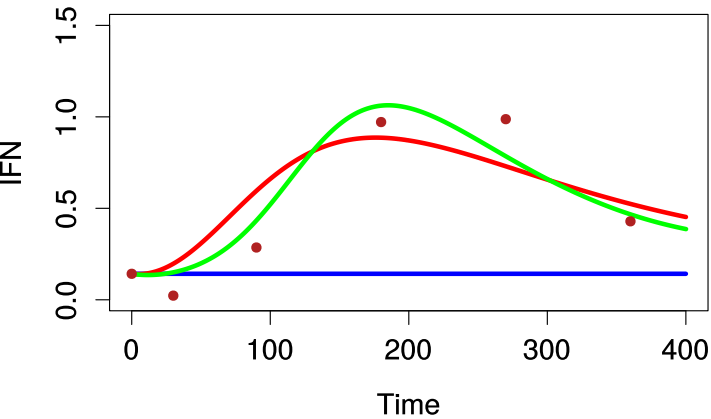**B**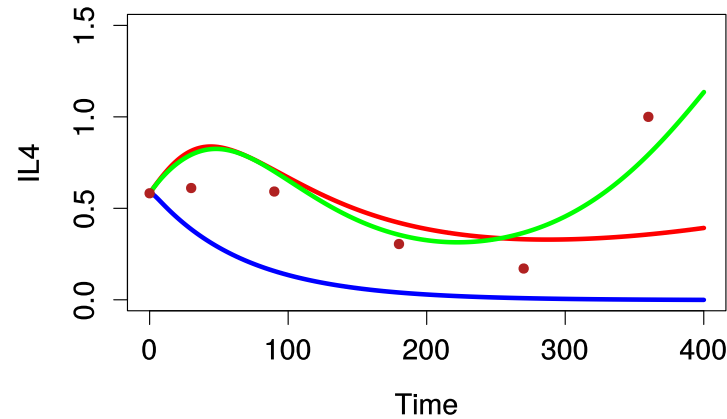**C**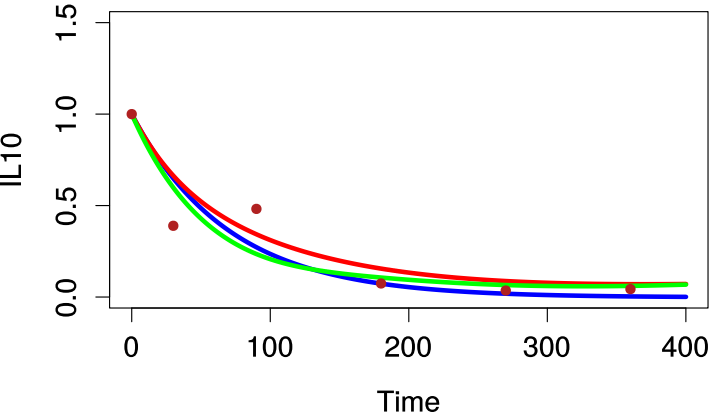**D**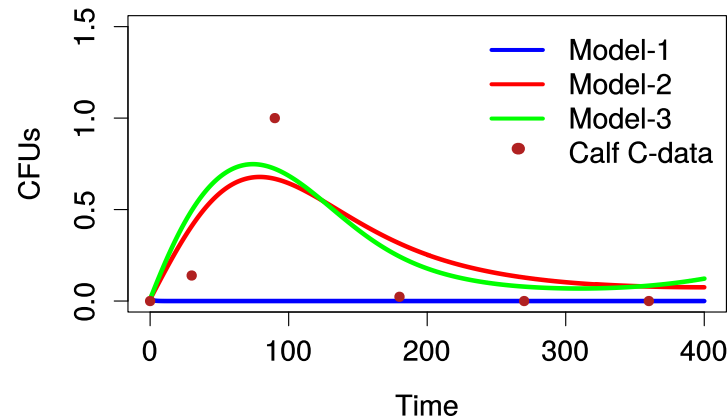

Supplement: S1 Fig — An illustration of model comparison and selection of models with close AIC values using visual examination or eyeballing. It is very clear that Model 1 fails to adequately explain all trends. However, the AIC values do no give enough information to select either Model 2 or Model 3. But by visually examining the fits, it becomes clear that Model 3 explains IL-4 and IFN-γ much better than Model 2 (A and B). These two models seem to explain IL-10 and CFU patterns in more or less a similar way (C and D). (PDF) [file pone.0141539.s001.pdf]

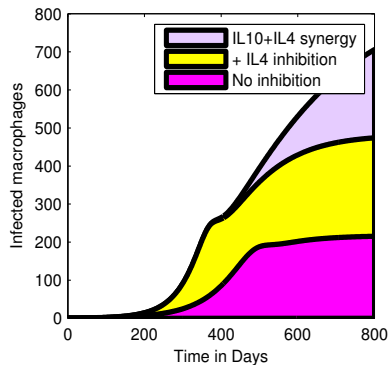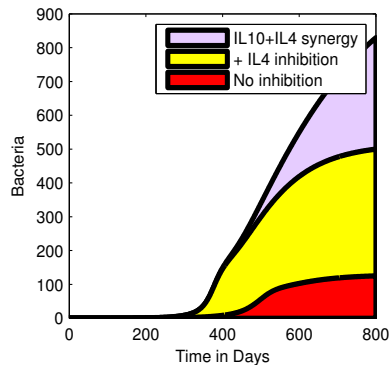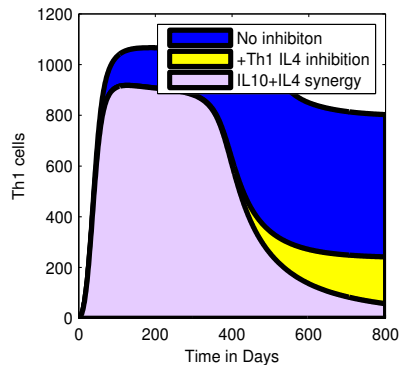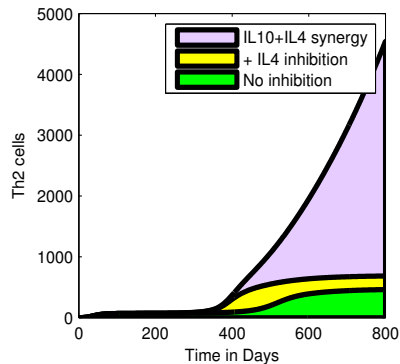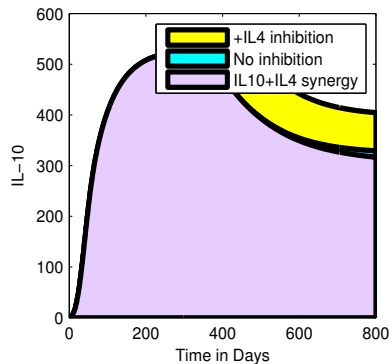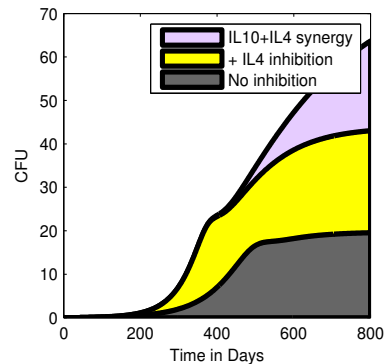

Supplement: S2 Fig — Simulations showing the effect of IL10- Th2 (IL4) synergy through (i) IL-10 enhancing Th2 cell expansion and (ii) Th2 suppressing the Th1 differentiation. Enhanced Th1 suppression is observed between the achieved suppression level of Th1 by the Th2 response only and the suppression level achieved through IL-10 and Th2 (IL-4) synergy. IL-10- Th2 (IL4) synergy was modelled using the terms (i) (θ1δmImTh01+b1Th2) (Th1 (IFN-γ) inhibition by Th2 (IL-4)) and (ii), α1Th2IL10(BB+MS) (Th2 proliferation), with b 1 = 0.005 and α 1 = 0.0002 while the rest of the model parameters are kept at the baseline values given in Table 2. The shading of cell populations before IL-10 inhibition are represented by the colours: grey-CFUs, blue-Th1 cells, red-bacteria, cyan-IL10, green-Th2 cells, pink-infected macrophages. The yellow shading represents the effect of IL-10 inhibition only, while the violet colour represents the effects of IL10 and Th2 synergy on the inhibition of Th1 expression. (PDF) [file pone.0141539.s002.pdf]

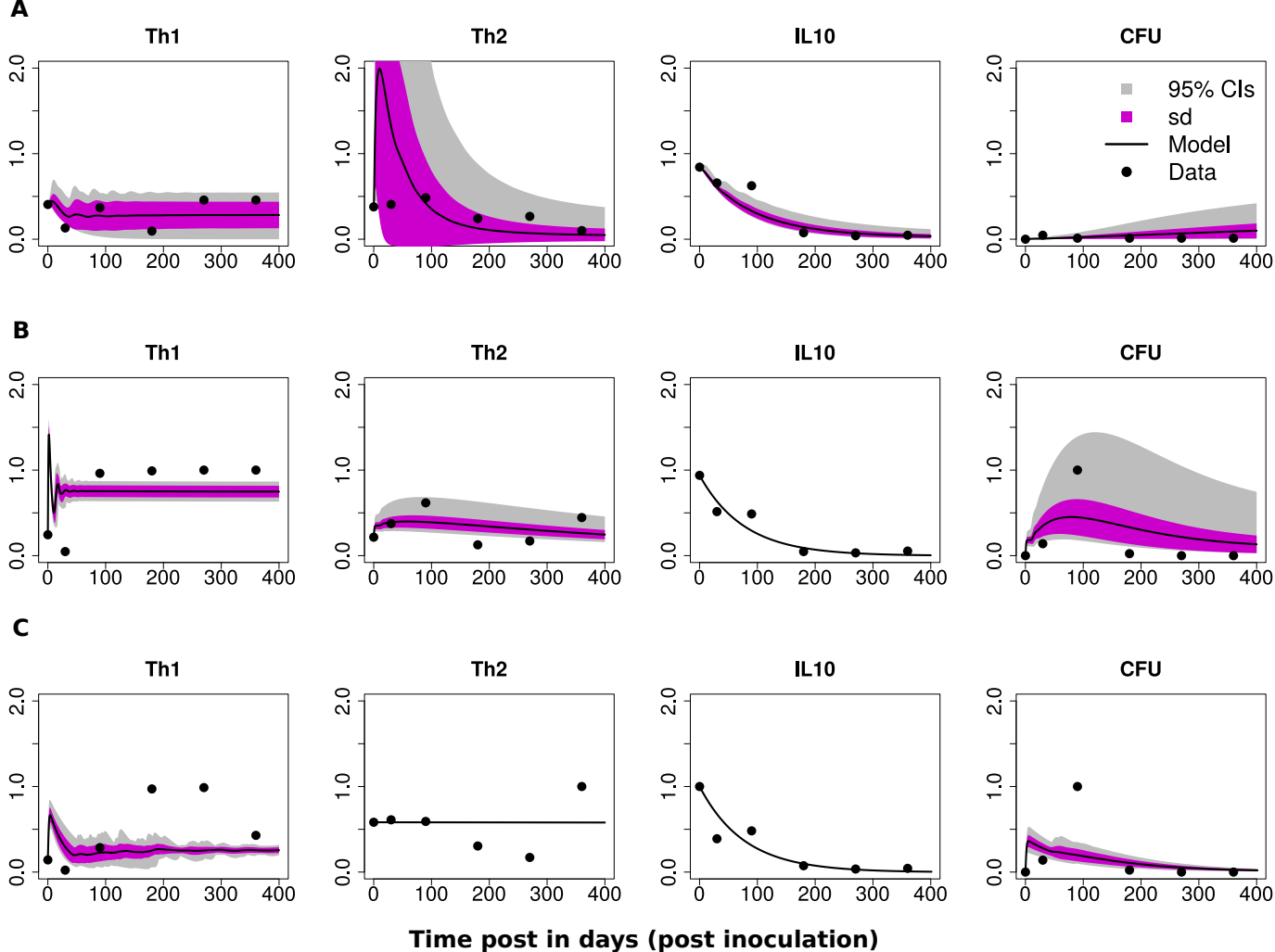

Supplement: S3 Fig — We show how fitting initial conditions, together with parameters alter the model fits. There is no much variation in the fitted model trajectories between S3 Fig, however, different parameters are estimated. The estimated parameters and initial conditions are given and their uncertainties are shown by the 95% CIs given in brackets. The shaded regions in this figure show the uncertainty in the model predictions that correspond to the estimated parameters (without including the uncertainty in the initial conditions). Calf A) k i = 0.0809(0.1466–0.0052), k b = 0.00780(0.01411–0.00071), λ 1 = 0.0123(0.0169–0.0103), δ m = 0.0074(0.0107–0.0050), δTreg = 0.1987(0.2909–0.1078), θ 2 = 216.85(412.45–104.75), δ B = 0.0803(0.0989–0.0496), M(0) = 0.0633(0.1296–0.0304), I m(0) = 0.4371(0.6397–0.2049), B(0) = 0.0234(0.0490–0.0012), Th 0(0) = 68.67(98.25–29.54). Calf B) k i = 0.5308(0.5682–0.3402), k b = 0.0207(0.0229–0.0168), λ 1 = 0.0962 (0.1056–0.0618), δ m = 0.4359(0.5200–0.3384), p 2 = 1.0306(1.1713–0.6776), μ 1 = 0.1861(0.2129–0.1208), δ B = 0.0367(0.0437–0.0251), μ CF = 0.0115(0.0127–0.0072), M(0) = 47.82(50.75–39.36), I m(0) = 6.5560(8.0265–4.2926), B(0) = 0.13801(0.1677–0.0935), Th 0(0) = 2.1774(2.3677–1.4161). Calf C) k b = 0.0076(0.0109–0.0052), λ 1 = 0.1290(0.1801–0.0844), δ B = 0.0115(0.0154–0.0081), a 1 = 0.0360(0.0674–0.0141), a 2 = 0.8066(1.1799–0.4908), μ 1 = 0.0359(0.0668–0.01139), μ 2 = 1.2369e-05(1.9019e-05-8.1854e-06), k i = 0.6753(0.9920–0.4471), M(0) = 329.43(545.35–183.93), I m(0) = 3.8573(8.4403–1.1546), B(0) = 0.5246(0.7719–0.3122), Th 0(0) = 3.7191 (5.1772–2.6257). (PDF) [file pone.0141539.s003.pdf]

**A**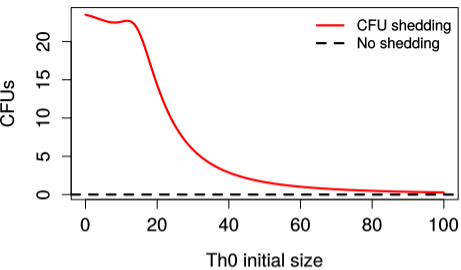**B**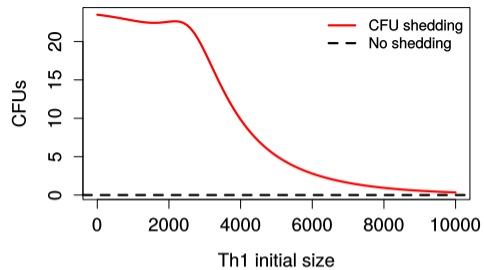**C**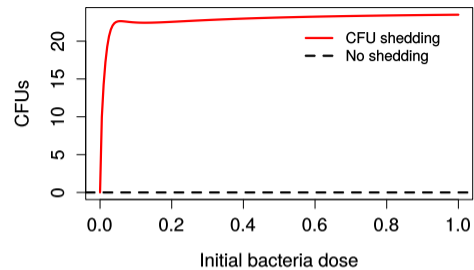**D**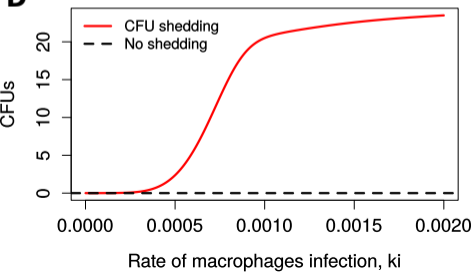**E**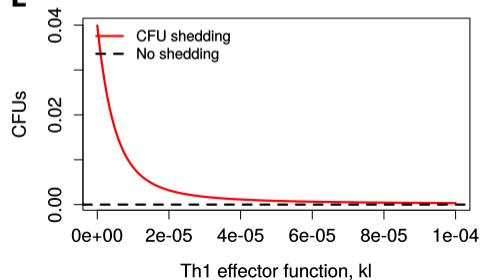**F**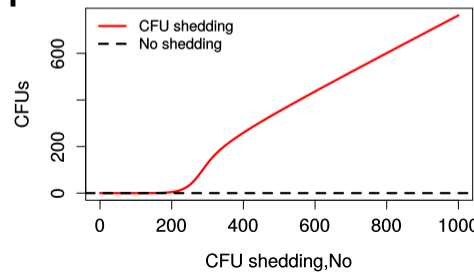

Supplement: S4 Fig — In panels (A, B and C) Initial conditions for bacteria dose, naïve T cells and Th1 cells were varied to evaluate how the steady state solutions of the model change. In panels (D, E and F) the rate of macrophage infection (k i), Th1 effector function (k l), and the infected macrophage burst size (N o) were varied to show how changing these parameters alter the disease (CFU shedding) endpoints. Increasing the Th1 effector function leads to a no shedding stable state, while increasing infection of macrophages and the amount of bacteria released at bursting leads to a stable CFU shedding state. Also, increasing the size of Th1 and Th0 cells leads to a no CFU shedding state, while increasing initial bacteria size exposure increases chances of disease, hence a stable CFU shedding state. (PDF) [file pone.0141539.s004.pdf]

**Th1**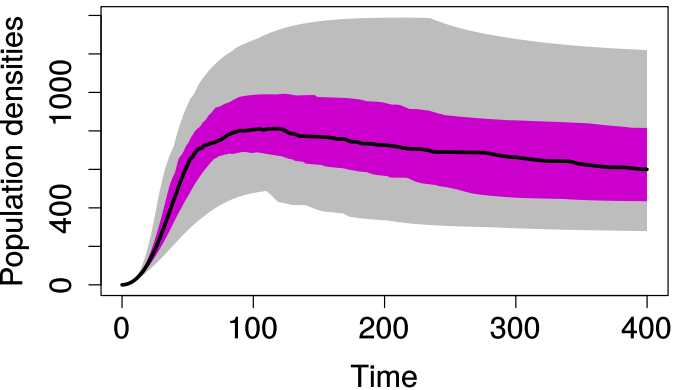**Th2**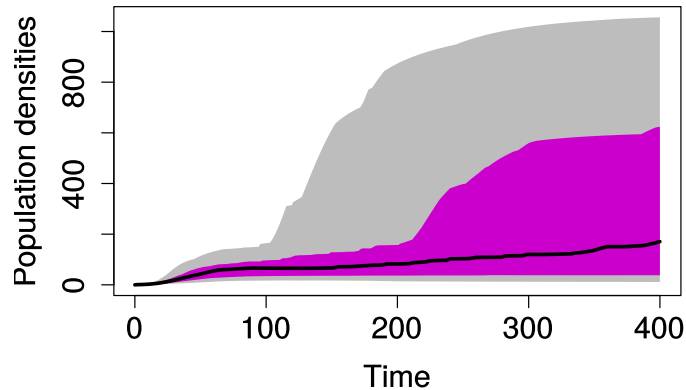**IL10**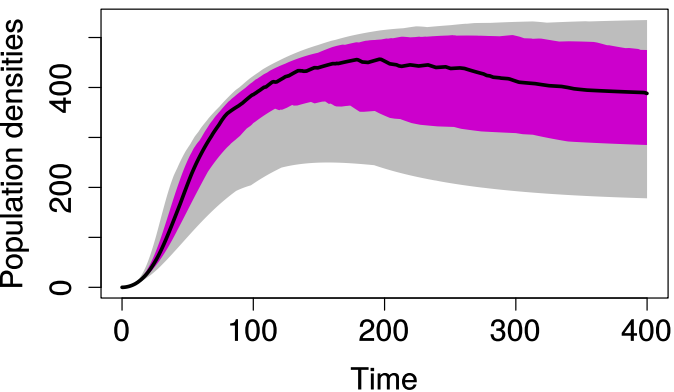**CFU**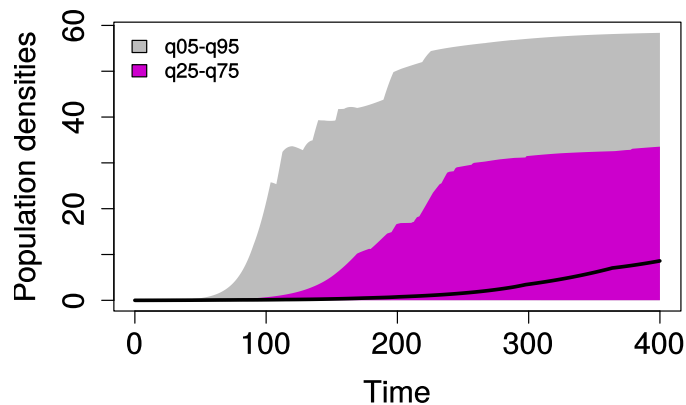

Supplement: S5 Fig — The model represented by system of Eq 2 was used to illustrate the model output sensitivity to variation in several model parameters (k i, k l, k m, k b, N o, δ B, λ 1, μ 1, μ 2, μ cf). The the uncertainty in model output variables is shown using grey (5th-95th quantiles) and magenta (25th-75th quantiles) coloured regions. (PDF) [file pone.0141539.s005.pdf]

**IFN**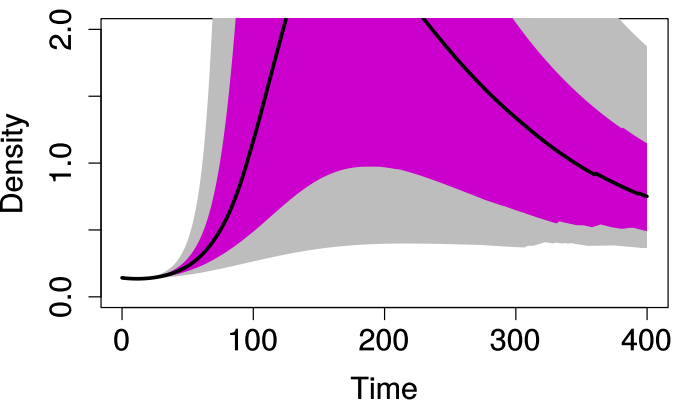**IL4**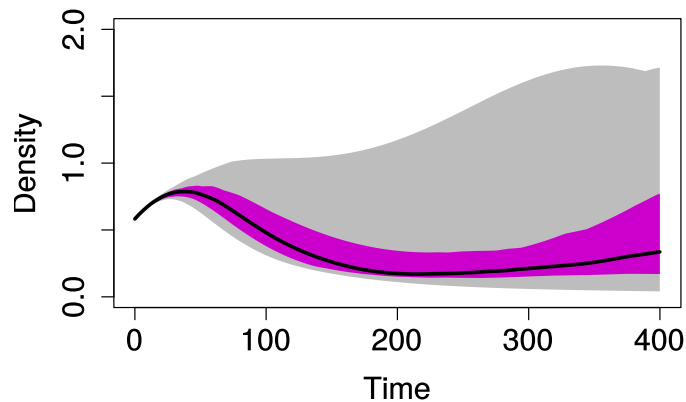**IL10**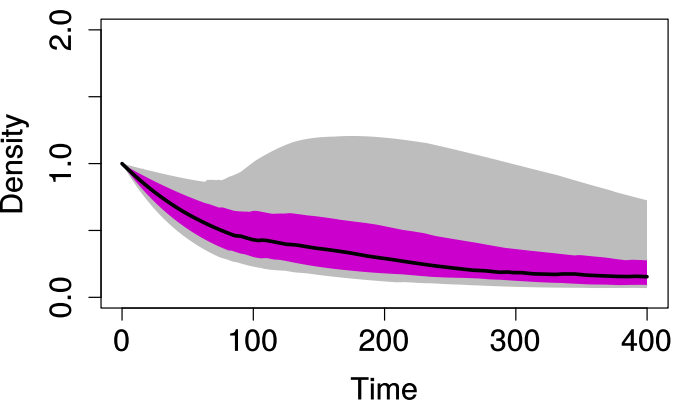**CFU**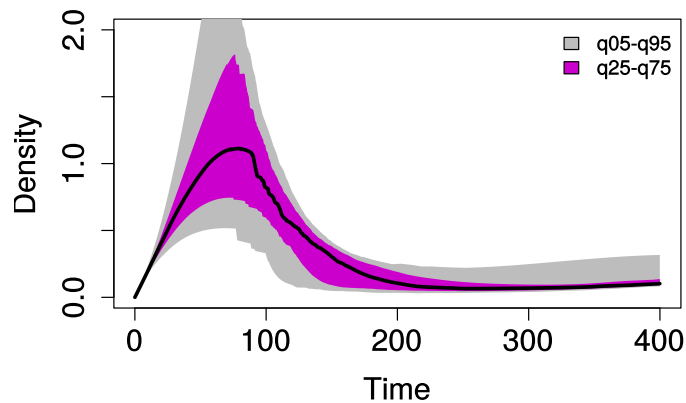

Supplement: S6 Fig — Model 3 (for calf C) was used to demonstrate the effects of varying several model parameters (α 1, α 2, α 3, α 4, μ 1, μ 2, μ 3, μ 4, r) to show the uncertainty in model output variability. The shaded regions show the 5th-95th quantiles (grey) and (magenta) 25th-75th quantiles, in the simulated trajectories. (PDF) [file pone.0141539.s006.pdf]
