# Supplementary material for: Predicting the Role of IL-10 in the Regulation of the Adaptive Immune Responses in Mycobacterium avium Subsp. paratuberculosis Infections Using Mathematical Models
Source: PLoS One. 2015 Nov 30;10(11):e0141539. doi: 10.1371/journal.pone.0141539 (PMC4664406; doi:10.1371/journal.pone.0141539)
Supplement: S1 Table — AIC values used to select the mechanistic terms used to model MAP CFU shedding in the cell compartmental model. The term λ 1 I m was selected because it is believed that MAP bacteria can escape from the intestine into the gut via infected cells. Also, there is no strong statistical information to select one term over the other. (DOCX) [file pone.0141539.s007.docx]

Table S1: **Selection of the shedding terms**

| Terms | AIC |
| --- | --- |
| $\lambda_{1}I_{m}+\lambda_{2}B$ | 18.3 |
| $\lambda_{1}I_{m}$ | 18.1 |
| $\lambda_{2}B$ | 16.9 |

AIC values used to select the mechanistic terms used to model MAP CFU shedding in the cell compartmental model. The term $\lambda_{1}I_{m}$ was selected because it is believed that MAP bacteria can escape from the intestine into the gut via infected cells. Also, there is no strong statistical information to select one term over the other.
