# Supplementary material for: Predicting the Role of IL-10 in the Regulation of the Adaptive Immune Responses in Mycobacterium avium Subsp. paratuberculosis Infections Using Mathematical Models
Source: PLoS One. 2015 Nov 30;10(11):e0141539. doi: 10.1371/journal.pone.0141539 (PMC4664406; doi:10.1371/journal.pone.0141539)
Supplement: S2 Table — Terms used to model the production of IL10 in the cell compartmental model and their corresponding AIC values. The term, θ 3 I m Th 0, was selected to model IL10 because of its associated AIC value. (DOCX) [file pone.0141539.s008.docx]

Table S2: **Selecting terms that are used to generate IL-10 in the models**

| Terms | AIC |
| --- | --- |
| $\theta_{3}I_{m}Th_{0}$ | 9.66 |
| $\theta_{3}BTh_{0}$ | 17.57 |
| $\theta_{3}Th_{2}Th_{0}$ | 16.90 |

Terms used to model the production of IL10 in the cell compartmental model and their corresponding AIC values. The term, $\theta_{3}I_{m}Th_{0},$ was selected to model IL10 because of its associated AIC value.
