# Supplementary material for: Predicting the Role of IL-10 in the Regulation of the Adaptive Immune Responses in Mycobacterium avium Subsp. paratuberculosis Infections Using Mathematical Models
Source: PLoS One. 2015 Nov 30;10(11):e0141539. doi: 10.1371/journal.pone.0141539 (PMC4664406; doi:10.1371/journal.pone.0141539)
Supplement: S1 Text — Mathematical models used to generate the predicted immune pathways that are shown in Fig 2B. S1 Text gives a brief explanation of the immune interactions that we predicted to be essential to explain the calf data and explains the simplifying assumptions of the empirical models. (DOCX) [file pone.0141539.s009.docx]

**Text S1: Cytokine signalling model equations**

The cytokine models are more empirical than the cell compartmental model and were designed to explain the data and predict potential interactions between the observed cytokines and CFUs without capturing all the essential biology. To do this we assumed that CFUs are a proxy of within cattle MAP bacteria, which is an oversimplification, since infected animals can express different immune responses, but may not excrete bacteria in their faeces. The limitation of this model is, however dealt with in the cell model, in which we tried to capture most of the essential MAP infection biology. Also, the cytokine model, does not explain the dynamics from the onset of infection, since it does not model the within animal bacteria density, but the excreted bacteria. Therefore, only explains the interactions that are important to reproduce the observed experimental data.

**Model 1 (Calf A)**

$$\frac{dIFN}{dt}=\alpha_{1}\frac{CFU*IFN}{(1+h_{1}IL_{4})(1+h_{3}IL_{10})}-\beta_{1}IFN,$$

$$\frac{dIL_{4}}{dt}=\alpha_{2}\frac{CFU*IL_{10}}{(1+CFU)}-\beta_{2}IL_{4},$$

$\frac{dIL_{10}}{dt}=\alpha_{3}IL_{4}-\beta_{3}IL_{10},$ (S1)

$$\frac{dCFU}{dt}=\alpha_{4}CFU*IL_{10}-\gamma_{1}IFN*CFU-\beta_{4}CFU.$$

This model predicts that MAP bacteria stimulates the expression of IFN-$\gamma$ while IL4 and IL10 inhibit the expansion of the IFN-$\gamma$ responses. IL4 is stimulated by the bacteria and the IL10, and IL4 induce the expression of IL10. Also, this model predicts IL10 to enhance bacteria multiplication hence shedding. IFN-$\gamma$ response are predicted to be protective.

**Model 2 (Calf B)**

$$\frac{dIFN}{dt}=\alpha_{1}\frac{CFU}{(A_{1}+IL_{4})}-\beta_{1}IFN,$$

$$\frac{dIL_{4}}{dt}=\alpha_{2}\frac{IL_{10}}{(A_{3}+IL_{10})(1+h_{2}IFN)}-\beta_{2}IL_{4},$$

$\frac{dIL_{10}}{dt}=\alpha_{3}IL_{4}-\beta_{3}IL_{10},$ (S2)

$$\frac{dCFU}{dt}=\alpha_{4}CFU*IL_{10}-\gamma_{1}IFN*CFU-\beta_{4}CFU.$$

Model 2 predicts MAP infection to stimulate IFN-$\gamma$ expression while IL4 inhibit this process. MAP bacteria and IL10 promote expansion of the IL4 responses while IFN-$\gamma$ suppress the IL4 stimulation. IL10 expression and CFU excretion are explained in a similar way as in Model 1.

**Model 3 (Calf C)**

$$\frac{dIFN}{dt}=\alpha_{1}IFN*CFU-\beta_{1}IFN,$$

$$\frac{dIL_{4}}{dt}=\alpha_{2}\frac{IL_{4}}{(1+h_{1}*IFN*CFU)}-\beta_{2}IL_{4},$$

$\frac{dIL_{10}}{dt}=\alpha_{3}IL_{4}*IFN-\beta_{3}IL_{10},$ (S3)

$$\frac{dCFU}{dt}=\alpha_{4}IL_{10}*IL4+r*CFU-\gamma_{1}IFN*CFU-\beta_{4}CFU.$$

In Model 3, IFN-$\gamma$ responses proliferate without inhibition from IL4 and IL10 mechanisms. However, the expansion of IL4 is inhibited by IFN-$\gamma$ responses. The expression of IL10 is supported by both IL-4 and IFN-$\gamma$ responses. MAP bacteria are predicted to expand while both IL4 and IL10 are predicted to enhance their multiplication, hence their excretion. IFN-$\gamma$ responses are predicted to have a similar role like in all the other models.
